# DNA virus–host patterns in lake and marine environments over the last glacial cycle

**DOI:** 10.1093/ismejo/wrag025

**Published:** 2026-02-19

**Authors:** Christiane Boeckel, Simeon Lisovski, Kathleen R Stoof-Leichsenring, Josefine Friederike Weiß, Sisi Liu, Lars Harms, Ulrike Herzschuh

**Affiliations:** Polar Terrestrial Environmental Systems, Alfred Wegener Institute Helmholtz Centre for Polar and Marine Research, Potsdam D-14473, Germany; Institute of Biology and Biochemistry, University of Potsdam, Potsdam D-14476, Germany; Polar Terrestrial Environmental Systems, Alfred Wegener Institute Helmholtz Centre for Polar and Marine Research, Potsdam D-14473, Germany; Polar Terrestrial Environmental Systems, Alfred Wegener Institute Helmholtz Centre for Polar and Marine Research, Potsdam D-14473, Germany; Polar Terrestrial Environmental Systems, Alfred Wegener Institute Helmholtz Centre for Polar and Marine Research, Potsdam D-14473, Germany; Institute of Biology and Biochemistry, University of Potsdam, Potsdam D-14476, Germany; Polar Terrestrial Environmental Systems, Alfred Wegener Institute Helmholtz Centre for Polar and Marine Research, Potsdam D-14473, Germany; Data Science Support, Alfred Wegener Institute Helmholtz Centre for Polar and Marine Research, Bremerhaven D-27568, Germany; Polar Terrestrial Environmental Systems, Alfred Wegener Institute Helmholtz Centre for Polar and Marine Research, Potsdam D-14473, Germany; Institute of Biology and Biochemistry, University of Potsdam, Potsdam D-14476, Germany; Institute of Environmental Science and Geography, University of Potsdam, Potsdam D-14476, Germany

**Keywords:** DNA viruses, sedimentary ancient DNA, paleoecology, marine ecosystem, terrestrial ecosystem

## Abstract

Viruses are integral to population dynamics, biogeochemical cycling, and host evolution, making them essential for ecosystem function. We explore long-term virus–host interactions mainly within microbial ecosystems in lake and marine environments across the late Pleistocene and Holocene. Sedimentary ancient DNA (sedaDNA) from five Siberian lakes and three Subarctic/Antarctic marine cores were analysed to infer past DNA virus taxa from metagenomic sequences. Viruses accounted for 357 161 reads (0.089% of total mapped reads), distributed across 2084 unique viral taxa. Virus communities differ between lakes and marine sites, with lakes dominated by *Caudoviricetes* and marine environments featuring *Caudoviricetes* and *Algavirales*. Each time series shows compositional changes from the Pleistocene to the Holocene, supporting sedaDNA as a tool to reconstruct time-resolved ancient viral assemblages. Among the most abundant viruses, we identified 83 virus–host pairs documented in published literature, spanning bacterial, archaeal, and eukaryotic hosts, and assessed their associations based on co-occurrence correlations. Over millennia, virus–host co-variations are particularly stable in marine systems, especially for phytoplankton-infecting viruses. However, in the Bering Sea, we find a lack of virus–host correlation, likely because an Arctic *Pelagibacter* strain expanded after the Bering Strait opened, potentially due to absent viral infection, although database limitations prevent clear interpretation. Antagonistic patterns also appear between bacteriophages and hosts, possibly linked to shifts between lytic and lysogenic cycles in response to environmental changes. This study demonstrates that sedaDNA time-series can reveal ancient viral community structures and long-term ecological patterns, highlighting the value of ancient viromes in understanding ecosystem-specific responses to environmental change.

## Introduction

Viruses, particularly those infecting microbes, are critical components of ecosystems but are often overlooked in biodiversity assessments. Their reliance on hosts for replication affects host fitness and can cause entire community shifts. Beyond influencing microbial host populations, viruses shape host evolution via gene transfer and are involved in biogeochemical cycling [1–3]. Understanding long-term virus–host dynamics is therefore crucial to predicting how ecosystems respond to environmental change.

Viruses infecting bacteria (bacteriophages) use host cellular machinery to reproduce, ultimately causing cell lysis. However, some bacteriophages enter a lysogenic life cycle in which they persist in the host cell and remain dormant until conditions favour viral production and a switch to lysis [4, 5]. Similar latent phases occur in some eukaryotic viruses [6, 7]. This dormancy allows viruses to form “seed banks” within ecosystems, re-emerging when conditions favour replication [8]. Periods of reduced viral production may also result from the evolutionary arms race between viruses and hosts, described by the Red Queen Theory. In this constant struggle, hosts evolve mechanisms to resist infection, whereas viruses adapt to overcome these defences, leading to suppressed viral replication and a temporary advantage for the hosts [9]. The mechanism of how potential host resistance or viral dormancy affects virus–host dynamics resulting in reduced virus productivity over long timescales is poorly understood.

Viruses show strong habitat specificity, with distinct communities found in different environments [10]. Yet within a given habitat, viral populations can be widely distributed, as seen in marine viruses dispersed by ocean currents [8, 10]. In contrast, freshwater virus communities, such as those in polar lakes, are more isolated and distinct [11]. Although these patterns are documented in modern systems, it remains unclear whether such ecological structures persist over geological timescales or how they respond to millennial scale, natural environmental changes.

Temperature correlates with viral community composition and infection dynamics in both marine [8, 12] and freshwater systems [11], and studies suggest that viral communities are susceptible to global warming [13, 14]. The last glacial–interglacial cycles were characterised by pronounced temperature variations of several degrees Celsius [15]. During the last glacial period, the Northern Hemisphere was extensively glaciated, reaching its maximum during the Last Glacial Maximum (26.5–19 ka BP, thousand years before present) [15]. Yet, despite the extremely cold climate, Northeast Siberia remained mostly ice-free [16]. Glaciation caused sea levels to drop by 120–130 metres [15], resulting in the exposure of a land bridge between Siberia and Alaska, known as Beringia, which disrupted water flow between the Arctic Ocean and the Northern Pacific. As global temperatures began to rise around 19 ka BP [15], progressive deglaciation and sea-level rise gradually submerged the land bridge, leading to the reopening of the Bering Strait around 11–12 cal ka BP [17–19]. A particularly intense period of warming began around 14 ka BP in the Northern Hemisphere, marking the transition from the last glacial period of the late Pleistocene towards the Holocene interglacial [20]. This led to phases of intense glacial meltwater releases and terrestrial input into the Bering Sea at that time [21–23]. Concurrently, the Southern Hemisphere experienced a temporary cooling between 14.7 and 12.7 ka BP, known as the Antarctic Cold Reversal [24]. During this period, atmospheric and oceanic temperatures dropped, expanding sea ice around the Antarctic continent [25]. Such major environmental changes in both the Northern and Southern Hemisphere likely shaped virus–host dynamics, but direct evidence of how viral communities responded is lacking.

Past virus–host interactions have to be inferred from environmental records. Well-preserved remains from vertebrates enable the investigation of ancient host-associated viruses. Ancient viruses have been identified in preserved faeces [26], bones [27], and soft tissues [28, 29]. These samples offer insights into viral interactions with animals, humans, and their respective microbiomes at the time of their decease. Ancient environmental biomes and their viral assemblages at the time have been inferred from ancient DNA in permafrost soil [30–33] and glacier ice [34, 35], some of which covered transitions between glacial and interglacial periods [30, 35]. However, reconstructions of ancient viral communities in aquatic systems and time-resolved records are missing so far.

In the last two decades, sedimentary ancient DNA (sedaDNA) has emerged as a reliable proxy to reconstruct ecosystem composition of the past across all domains [36, 37]. DNA from local and catchment aquatic and terrestrial organisms is buried and preserved in the sediments. Marine sedaDNA has previously been used to target specific virus–host systems such as *Emiliania huxleyi* and its *Coccolithovirus* [38]. In contrast, metagenomic shotgun sequencing of stratified sedaDNA provides the opportunity to reconstruct past viral assemblages and their potential hosts within the same samples through time [30]. However, whether such metagenomic sedaDNA data can be reliably used to reconstruct entire ancient viral communities and reveal long-term virus–host dynamics remains untested.

Here, we investigate the ancient DNA virus community preserved in sediments from marine and lake environments across the glacial–interglacial transitions of the past 124 000 years. Using metagenomic data from eight sediment cores spanning Subarctic, Arctic, and Antarctic sites, we aim to elucidate the spatial and temporal patterns of ancient viral communities. Specifically, we (i) compare viral assemblages between lake and marine systems and across the last glacial and current interglacial, and (ii) examine virus–host co-occurrence patterns to investigate ecological interactions and potential perturbations in the past. In this study, we demonstrate that sedaDNA enables the reconstruction of ancient viral communities and virus–host dynamics in both lake and marine environments over millennial timescales. We identified distinct differences in viral community composition between lakes and marine systems, with environmental context (lake vs. marine) exerting a stronger influence than geological epoch. Temporal patterns in viral taxa reflect major climatic transitions, particularly the shift from the last glacial period to the warmer Holocene. Additionally, we find both positive and negative long-term correlations between viruses and their hosts, suggesting that periods of reduced viral production, potentially driven by environmental change, host resistance, or viral dormancy, shaped virus–host interactions over time.

## Materials and methods

### Sediment cores and DNA extraction

In this meta-analysis, five Siberian lake sediment cores (Levinson-Lessing, Lama, Ilirney, Ulu, Bolshoe Toko) were compared to three marine cores from the Northern Pacific (SO201–2-KL77, SO201–2-KL12) and the Bransfield Strait, Antarctic Peninsula (PS97/072–01). These cores generally span the Pleistocene–Holocene, with the oldest record dating back to 124 cal ka BP. All DNA work was carried out in dedicated palaeogenetic DNA facilities at the Alfred Wegener Institute in Potsdam, using strict contamination precautions. The cores were originally collected for earlier palaeoecological studies, and their collection procedures, dating, and age–depth models are described in detail in previous publications (Table 1).

**Table 1:** Metadata of the sampled sites.

Environment	Site	Coordinates	Core name	Time period covered (age cal ka BP)	Additional references
Marine	KL77	56.33°N, 170.70°E; −2135.0 m (Shirshov Ridge, Western Bering Sea, North Pacific)	SO201–2-77KL	1.82–124.0 [39]	[21, 39–43]
	KL12	53.99°N, 162.37°E; −2173.0 m (Western Continental Slope off Kamchatka, Western Bering Sea, North Pacific)	SO201–2-12KL	1.08–19.9 [44]	[21, 39, 40, 44, 45]
	PS97	62.01°S, 56.06°W; −1992.9 m (East Bransfield Strait, Southern Ocean)	PS97/072–01	0.1–13.9 [46]	[21, 47]
Lake	Levinson-Lessing	74.27°N, 98.39°E; 48 m a.s.l. (Taymyr peninsula, Northern Siberia)	Co1401	0–58.2 [48]	[48–53]
	Lama	69.32°N, 90.12°E; 53 m a.s.l (Putorana Plateau, Central Siberia)	PG1341	0.1–23.0 [52]	[49, 54–56]
	Ilirney	67.56°N, 168.49°E; 421 m a.s.l (Anadyr Mountains region, Chukotka)	EN18208	0.1–54.6 [57]	[49, 58, 59]
	Ulu	63.34°N, 141.03°E; 950 m a.s.l (Oymyakon Upland, Upper Indigirka Basin, Northeastern Yakutia)	EN21103	2.74–43.2 [60]	[49]
	Bolshoe Toko	56.15°N, 130.30°E; 903 m a.s.l (northern slope of eastern Stanovoy Range, Southeastern Yakutia)	PG2133	1.43–33.6 [61]	[49, 52, 61–64]

The sedaDNA was extracted using the DNeasy PowerMax Soil Kit (Qiagen, Germany) with a modified protocol including proteinase K and DTT incubation, as previously described [65]. In total, 418 samples and 133 extraction blanks were produced. After DNA extraction, samples and blank controls were concentrated and purified using the GeneJet PCR Kit (ThermoFisher) and 10 μL of a 3 ng/μL solution of concentrated sample sedaDNA (and 10 μL DNA from the extraction blanks) were used as input to the single-stranded library preparation protocol [66, 67]. Each library batch contained, on average, six sedaDNA samples, one extraction blank, and one library blank. Library preparation and shotgun sequencing were performed as previously described [37, 49, 68]. Further details on blanks and sample read statistics are available (Supplementary Table S1).

In total, the dataset contains 35 extraction blanks and 40 library blanks. Potential contamination was evaluated by comparing taxonomic assignments in blanks and samples (Supplementary Table S2). For bacterial, archaeal, and eukaryotic taxa the proportion of reads in blanks relative to total reads from blanks and samples was consistently low (<0.2%). For viral taxa, 48 blanks were clean and 27 blanks showed 45 reads in total (0.013% from total viral reads from blanks and samples). Based on these patterns and the low contributions of blanks relative to the dataset, no taxa were excluded from the sample dataset.

### Bioinformatic pipeline and taxonomic assignment

The raw sequencing data (13 615 653 606 reads) were processed using a customised version of the “HOLI” pipeline [69] with a larger customized taxonomic reference database as previously described [37] which has been extended by additional virus reference genomes [49]. In short, quality checked reads were deduplicated before and after trimming from adapters, followed by alignment against a custom database using Bowtie2 [70]. The taxonomic classification was performed using metaDMG [71] and ngsLCA [72]. The alignment-based HOLI approach enables short-read taxonomic classification suited to the highly fragmented nature of sedaDNA, allowing reliable identification of both abundant and low-abundance taxa. The custom reference database was generated from RefSeq [73] and NT January 2021 databases from NCBI [74], PhyloNorway contigs [62], Bacterial and Archaeal genomes of GTDB v220 [75], high-quality virus genomes of IMG/VR v4.1 [76], and selected genomes from taxa of the Siberian (sub)arctic. Comprehensive information on all reference sources, including the additional Siberian (sub)arctic genomes, is available at https://github.com/sisiliu-research/EnviHoli.

### DNA damage pattern analysis and authentication

To ensure that the reconstructed viral and microbial communities represent ancient rather than modern DNA, we assessed post-mortem DNA damage patterns. This authentication step exploits characteristic signatures of ancient DNA, such as elevated cytosine-to-thymine (C-to-T) substitutions at fragment ends. Damage analysis followed a published pipeline [54, 77] in which quality-filtered, error-corrected metagenomic reads were de novo assembled into contigs, aligned, and processed to estimate damage profiles. Contigs were taxonomically classified with Kraken2 [78] against the nt database (downloaded in October 2022), and damage estimates were merged by contig ID. We then applied PyDamage [79] to statistically model C-to-T substitution frequencies and identify contigs carrying authentic ancient DNA patterns for viruses and bacteria. Specifically, PyDamage fits an end-enriched decay of C-to-T along reads and tests it against a flat null using a binomial likelihood-ratio framework [79]. Contigs were retained if the prediction accuracy was ≥0.6 and the length ≥ 1000 bp. 5′ C-to-T substitution frequencies were summarised by taxonomic group, and reads mapping to these retained contigs were considered as potentially ancient.

### Data analysis

All subsequent data analyses were performed using R (v4.3.2; https://www.r-project.org/) with tidyverse (v2.0.0) [80]. Unless otherwise stated, plots were generated using ggplot2 (v3.5.1) and cowplot (v1.1.3; https://wilkelab.org/cowplot/). The maps with the coring locations were generated using sf (v1.0–16) and rnaturalearth (v1.0.1). Plot alignments were done using Affinity Designer (v1.10.8).

To process taxonomic count data, we followed a previously published tutorial for community analysis of the EnviHoli pipeline [49], including filtering of taxa of interest and aggregation of reads assigned to lower taxonomic ranks. The ngsLCA output is provided as read counts assigned to individual taxa, where reads attributed to higher taxonomic levels do not include those assigned to subordinate taxa. Hence, for each sample, reads assigned to lower-level taxa (species, genus, family, order, class, phylum) were cumulatively aggregated, yielding read counts at all taxonomic levels. To account for differences in sequencing depth, aggregated taxonomic count data were normalised by total sum scaling, yielding relative abundances.

Rarefaction analysis was performed to evaluate the effect of sequencing depth on the distribution of viral taxa between environments. Reads were subsampled to depths ranging from 10^2^ to 10^8^ in successive log-scale steps using the *rrarefy* function of the vegan package (v2.6.4; https://vegandevs.github.io/vegan/). At each depth, the proportions of lake-unique, marine-unique, and shared viral species were calculated, and the number of distinct species was recorded.

To explore the overlap of taxonomic diversity across sediment cores, virus species lists were extracted for each core and compared and visualised in an upset plot using ggVennDiagram (v1.5.3) [81]. In total, 94.7% of viral reads could be assigned to the species level.

Community structure was further assessed using non-metric multidimensional scaling (NMDS) based on Bray–Curtis dissimilarities of Hellinger-transformed [82] relative abundances of the virus species. Redundancy analysis (RDA) and partial RDA were performed on Hellinger-transformed relative abundances using the vegan package to evaluate the contribution of environmental variables and geological epochs on the variance of the community composition. Statistical significance of the constrained ordinations was assessed using permutation-based ANOVA as implemented in the *anova.cca* function from the vegan package and 1000 permutations.

Pairwise similarity between sediment cores was quantified by binning samples to the overlapping time interval between each core pair using a bin width defined as the maximum median sampling interval of the two cores. Within each time bin, viral species relative abundances were averaged per core, transformed using the Hellinger transformation, and the Euclidean distance between the two cores was calculated. Pairwise core dissimilarity was defined as the mean Hellinger distance across all shared time bins and visualised as a heatmap. To assess the influence of highly dominant viral taxa on similarity patterns, pairwise core dissimilarities were additionally calculated after excluding the most abundant viral taxon in lakes (“uncultured Caudovirales phage”) and in marine cores (Virus NIOZ-UU157) within the whole dataset. The heatmap of the unfiltered similarity analysis is available (Supplementary Fig. S6).

Temporal patterns in virus community composition were visualised using stratigraphic area plots, with relative abundances with respect to viral reads plotted against calibrated ages. Stratigraphic summaries in the Supplementary included only taxa present across all cores of a given environment or those reaching high relative abundance in at least one core, shown only for the cores where they were abundant. The selected taxa showed obvious temporal patterns by visual inspection.

To assess long-term virus–host co-occurrence patterns, we performed correlation analyses based on cumulative relative abundances, calculated with respect to all reads. Viruses were associated with their host based on published literature and databases (Supplementary Table S3). Viral species were grouped according to their inferred host groups, and their relative abundances were summed accordingly. For host taxa, eukaryotic hosts were summarised at the phylum level, whereas bacterial hosts were summarised at the genus level. This taxonomic resolution was chosen based on the assumption that eukaryotic viruses often exhibit broader host ranges than bacteriophages.

In a complementary analysis focused specifically on bacteriophage–bacterium dynamics, viruses were grouped according to the bacterial class of their known or inferred host. Host information was derived from a curated virus–host pairing list (Supplementary Table S3), supplemented by the Virus Metadata Resource by ICTV (VMR/MSL38 v2; https://ictv.global/news/vmr_release_0923). When virus–host associations were not explicitly listed, we used the trivial virus names, which typically include the host genus followed by “phage,” to infer host identity. Viral genera were then assigned to host bacterial classes based on their known host genus. Correlations were calculated using Spearman’s rank correlation, and significance of correlation coefficients was assessed using one-sided Wilcoxon signed-rank tests.

## Results and discussion

### Ancient viruses in lake and marine environments

**Figure 1:**
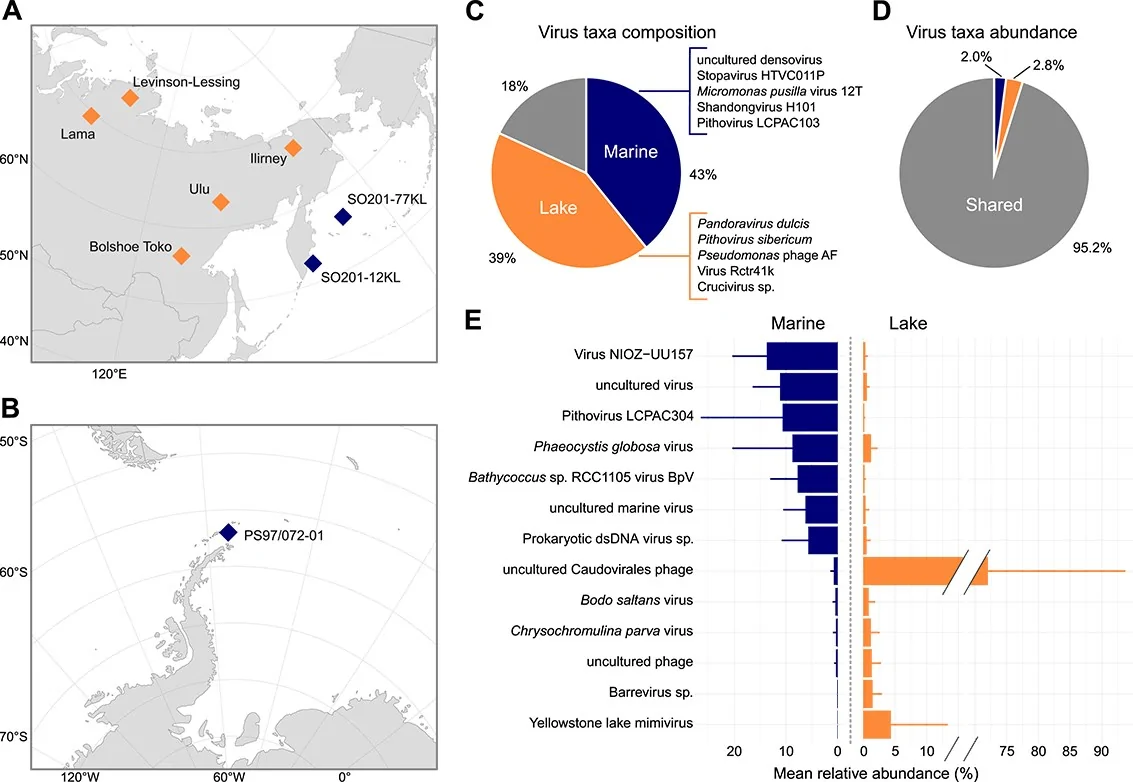
Site locations and virus species overlap. Map of the coring sites in the (A) Northern and (B) Southern Hemisphere. (C) Proportion of distinct viral species unique to marine or lake environments or shared between both. The top five viral species by mean abundance are listed for each environment. (D) Proportion of reads assigned to virus species unique to marine or lake environments or shared between both. (E) Bar plot of mean relative abundance (± standard deviation) of the most abundant shared taxa. Colours indicate lake (orange), marine (blue), and shared (dark grey) environments. Host genus and species names contained within virus names are shown in italics for consistency.

We retrieved a total of over 13 billion (13 615 653 606) paired-end reads from 254 lake and marine sediment samples and 75 blanks across eight sites (Fig. 1A). Of these, 400 186 289 reads (2.9%) aligned to eukaryotic, bacterial, archaeal, or viral DNA genome references of our custom database. Viruses accounted for 357 161 reads (0.089% of all mapped reads), across 2084 identified virus taxa with at least one read and a median of 47 virus taxa and 396 reads per sample. Of all viral reads, 94.7% were assigned at species level, representing 65.3% of the virus taxa, whereas ~4% could not be subclassified within the Virus realm. The remaining viral reads were assigned to genus level or higher.

Viral and bacterial contigs exhibited characteristic patterns of ancient DNA damage, with elevated C-to-T substitutions at fragment ends caused by cytosine deamination. We assessed post-mortem damage patterns using PyDamage [79] for viruses and bacteria. Although GC content can vary strongly between species of both viruses and hosts, benchmarking of PyDamage has shown that GC content does not affect its predictions [79]. We filtered contigs with a prediction accuracy ≥ 0.6 and length ≥ 1000 bp and plotted 5′ C-to-T substitution frequencies for each taxonomic group. Reads mapping to these retained contigs were considered as potentially ancient. Average rates at the first nucleotide position ranged from 0.12–0.19 for viruses and were comparable to those seen in bacterial hosts (0.11–0.19). Among damaged reads, a pronounced decline in C-to-T frequency across the first 10 read positions was observed (Supplementary Fig. S1 and S2), consistent with expected post-mortem damage accumulation over time. The C-to-T frequency was generally lower at site PS97, a pattern also observed in other taxa from this site [47], likely reflecting better overall DNA preservation in this sediment core. Taken together, the high number of ancient contig assignments and consistent damage patterns support the conclusion that both viral and host DNA are indeed ancient.

**Figure 2:**
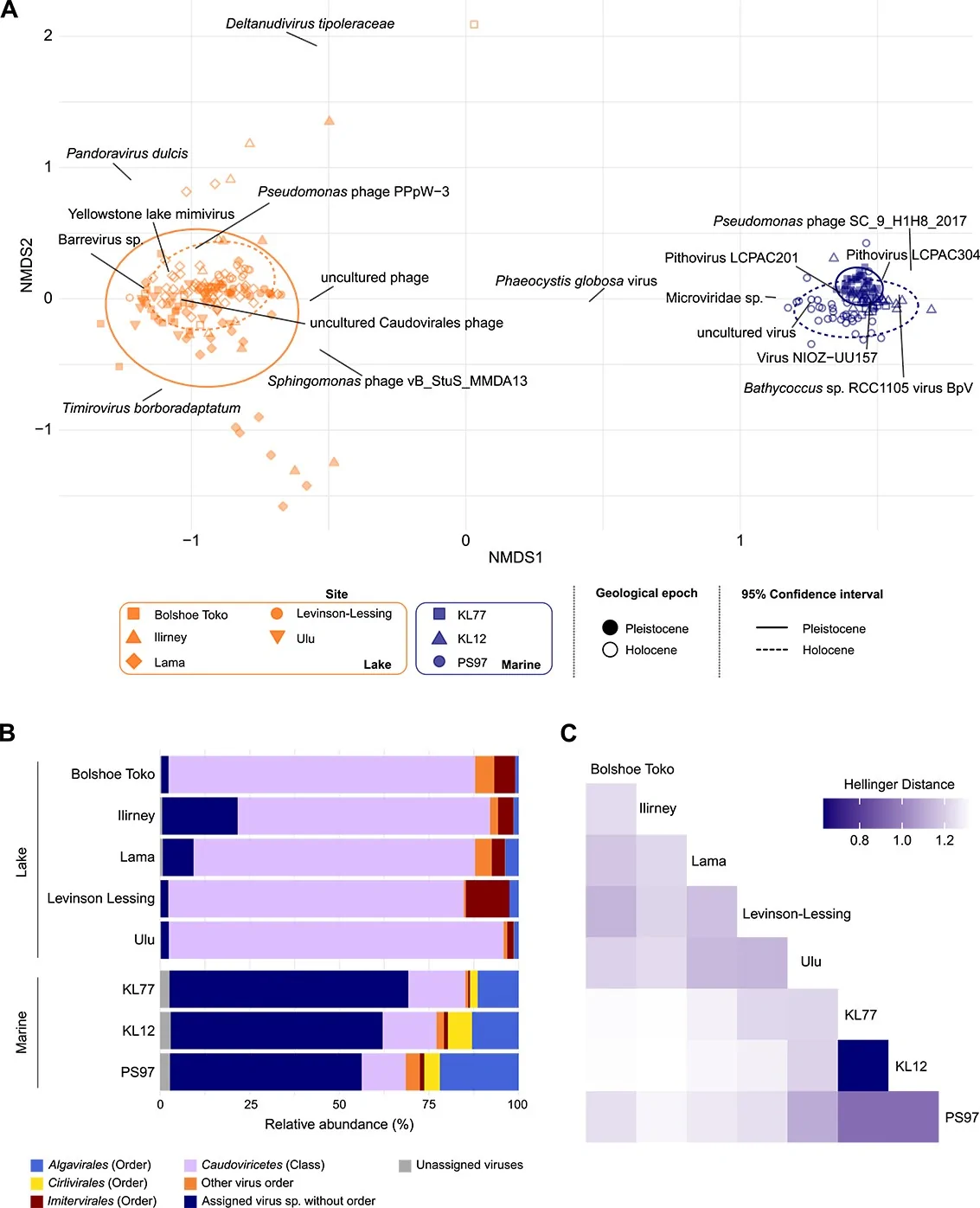
Virus community composition. (A) Non-metric multidimensional scaling (NMDS) plot of virus species composition coloured by environment: marine (blue) and lake (orange). Individual sites are distinguished by shape; Pleistocene samples are filled, and Holocene samples are empty. Ellipses represent the 95% confidence intervals for Holocene (dashed line) and Pleistocene (solid line) samples. Loadings of the 16 most abundant virus species are labelled. Host genus and species names contained within virus names are shown in italics for consistency. (B) Composition of ancient virus orders in lake and marine cores. The three most abundant virus orders are coloured light blue, yellow, and red. *Caudoviricetes* (aggregated at the class level) are shown in purple. All other orders are grouped and shown in orange. Viruses classified below the superkingdom level but unassigned to an order are in dark blue. Viruses not classified within the virus superkingdom are shown in grey. (C) Heatmap of pairwise Hellinger distances of the virus species community between sites after exclusion of the dominant taxa “uncultured Caudovirales phage” and virus NIOZ-UU15. Darker shading indicates higher similarity.

Many virus species were unique to either lake (758; 43%) or marine (697; 39%) sediment cores, with only 323 species (18%) shared between the two environments (Fig. 1C). A rarefaction analysis showed that these proportions were broadly consistent across several orders of magnitude of sequencing depth (Supplementary Fig. S3). These unique taxa were rare, comprising just 4.8% of viral reads, whereas the shared species accounted for 95.2% but were typically dominant in only one environment (Fig. 1D and E). Both non-metric multidimensional scaling (NMDS) and RDA indicated separation, with samples clustering by environment (Fig. 2A) and environment alone explaining 0.6% of the variance (*P <* .05). This is consistent with modern aquatic viromes in which marine and freshwater systems share only a small fraction of virus taxa [10]. This pattern may reflect the rarity of marine–freshwater transitions for viruses and their hosts, due to osmotic barriers, competition, and predation by locally adapted species [83].

Beta-diversity analyses revealed differences between Holocene and Pleistocene samples, as visualized by NMDS ordination based on radiocarbon-dated age (Fig. 2A). However, RDA indicated that epoch explained only a very small fraction of the variance and did not reach statistical significance (adjusted *R*^2^ = 0.01, *P* = .064). Similar differences between glacial and interglacial virus communities have been observed in ice core studies [35], although one permafrost study reported no strong differences, likely due to coarser taxonomic resolution [30]. In modern samples, polar and temperate viral communities differ substantially in both lakes [11] and oceans [8, 12]. Hence, the observed epochal differences here are likely linked to the temperature gradient between the geological epochs. Taken together, these patterns are consistent with environmental drivers in modern systems [8, 10–12], indicating that the sedaDNA-derived viral assemblages capture meaningful ecological variation through time.

Each environment was characterised by a distinct viral community. Lake samples were dominated by *Caudoviricetes*, which infect bacteria and archaea, and many viruses belonged to *Imitervirales*, known to target amoebae (Fig. 2B). A previous study similarly found *Caudoviricetes* enriched in lake sediments compared to surface waters and suggested that this may reflect better preservation of tailed phages in sediments [84]. In marine samples, a large proportion of viruses could be assigned at the species level but remained unclassified in the current ICTV taxonomy. Among classified groups, *Caudoviricetes* and *Megaviricetes* were most abundant. The latter includes *Algavirales* and *Imitervirales*, which target algae and protists, respectively, consistent with modern marine samples [1]. *Cirlivirales*, a group with a broad host range including chordates, crustacea, and possibly seaweed [85, 86], were more abundant in marine samples (median = 3.41%) than in lake samples (median = 0.29%).

In 86% of lake samples (132 of 154), over half of viral reads were assigned to NCBI Taxonomy ID 2100421, labelled as “uncultured Caudovirales phage”. This group, comprising over 4000 environmental DNA sequences classified within the *Caudoviricetes* class, is predominantly associated with freshwater environments. It was highly dominant (>90% of viral reads) in 24.7% of lake samples but reached only 4% in marine samples (Supplementary Fig. S4). Given that only two sequences in this group originated from estuarine marine contexts, their presence in marine sediments likely reflects allochthonous input from terrestrial and freshwater sources, similar to patterns observed for plant sedaDNA [21]. Many of these unclassified *Caudoviricetes* likely also include bacteriophages infecting cyanobacteria, consistent with the ecological importance of cyanophages in aquatic environments [87].

Ancient marine viral communities showed greater similarity across sites than lake viral communities (Fig. 2C). In particular, the two North Pacific cores KL12 and KL77 were highly similar, which coincided with a high number of shared virus taxa (Supplementary Fig. S5). In contrast to the marine cores, which shared 94 virus taxa, the lakes exhibited more distinct virus communities, with only 23 shared taxa (1.1%). Each lake harboured 66–226 unique virus taxa, more than were shared with other lakes (e.g. 42 shared between Lakes Lama and Levinson-Lessing). However, this pattern was only evident after excluding the highly dominant taxon labelled “uncultured Caudovirales phage” from the similarity analysis. By contrast, removal of the most abundant marine viral taxon (Virus NIOZ-UU157) had little effect on marine similarity patterns (Fig. 2C and Supplementary Fig. S6). This mirrors high virus endemism observed in contemporary polar lakes, likely driven by limited connectivity [11], and parallels elevated host endemism in polar and high-elevation lakes [88]. The strong similarity between marine cores, particularly those in close proximity, reflects the connectivity of global ocean water masses and suggests viral dispersal via ocean currents, as has been demonstrated in modern viromes [8, 10].

### Host-driven differences in ancient viral community structure across marine environments

Virus composition differed considerably between the Antarctic core PS97/72–1 and the subarctic Pacific cores SO201–2-12KL and SO201–2-77KL (hereafter PS97, KL12, and KL77). In the NMDS analysis, samples from PS97 were spatially separated from KL12 and KL77 (Fig. 2A). The distinct virus community in PS97 was driven by a high relative abundance of alga-infecting viruses, whereas KL77 and KL12 contained more viruses infecting bacteria. The abundance of alga-infecting viruses in PS97 coincided with a high relative abundance of algae [81], resembling contemporary Southern Ocean communities [89].

During the sea-ice-rich Antarctic Cold Reversal, PS97 was dominated by *Pithovirus* LCPAC304 (mean relative abundance: 21.2%), which declined to 0.96% after the ACR. This species is unclassified, and its host is unknown. It is described as Pithovirus-like due to sequence similarity with *Pithoviridae* genes [90], suggesting it may infect *Pithoviridae* hosts such as amoebae or other protists.

After the Antarctic Cold Reversal and a decline in seasonal sea ice, the Haptophyta-infecting genus *Prymnesiovirus* became dominant (Supplementary Fig. S7). This shift coincided with a transition from a Haptophyta-dominated to a *Chaetoceros*-dominated diatom community, based on analyses of the same DNA extract [47]. Sea ice and temperature have been identified as potential drivers. Although the role of viral infection was not tested, *Prymnesiovirus* activity may have contributed to establishing a new steady state after the Antarctic Cold Reversal.

Around 8 cal ka BP, *Prasinovirus* became abundant, coinciding with an increase in its host *Bathycoccus prasinos* and a shift towards warmer open-ocean conditions [46]. In modern samples, *B. prasinos* is associated with polar waters but is less abundant in temperate regions [91]. However, growth may have been restricted in the colder conditions that prevailed in the Bransfield Strait before 8 cal ka BP [46].

In contrast to the Antarctic core PS97, *Prasinovirus* and *Prymnesiovirus* were abundant in KL77 and KL12 throughout the late Pleistocene but declined at around 12 and 14 cal ka BP, respectively (Supplementary Fig. S8 and S9). Prior to the Last Glacial Maximum, the coldest period of the late Pleistocene, KL77 also had high abundances of *Pelagivirus* and *Stopavirus*, both of which infect *Pelagibacter*, a member of the ubiquitous SAR11 clade. With the onset of the Holocene (~14 cal ka BP), *Kyanoviridae*, including *Llyrvirus* and *Mazuvirus*, both targeting *Synechococcus*, became abundant. These findings mirror the higher abundance of *Pelagibacter*-infecting and *Synechococcus*-infecting viruses in cold and warm habitats, respectively [92].

### Temporal shifts in lake virus communities since the last glacial maximum

Temporal changes in lake viral communities were highly variable across sites. Although many abundant taxa were present across multiple sites, their temporal patterns differed markedly, even in lakes that are geographically close or share climatic zones (Supplementary Figs S10–S14). Despite site-specific differences and the persistent dominance of *Caudoviricetes*, all lakes exhibited temporal shifts from the late Pleistocene to the Holocene. During the Pleistocene, *Casjensviridae* were detected in all lakes except Levinson-Lessing, probably being derived from the catchment, as their hosts can be associated with terrestrial plants (Supplementary Figs S10–S14). Although relatively rare (1.0%–4.6%), they occurred only before ~10 cal ka BP, making them the only virus family more abundant during cold glacial periods than during warm interglacial periods. Their absence in Lake Levinson-Lessing may be due to even colder conditions that suppressed host presence or viral transmission. The dominant genus, *Salvovirus*, infects *Xylella*, a widespread plant pathogen that relies on insect transmission and is positively associated with higher temperatures [93]. *Xylella* was present in all lakes during the glacial period, indicating cold tolerance, but elevated *Casjensviridae* abundance during this time implies that infection may have been favoured under environmental stress conditions. The Holocene saw a rise in alga-infecting *Phycodnaviridae*, especially in Lakes Levinson-Lessing and Ilirney (Supplementary Figs S10–S14), likely reflecting enhanced aquatic productivity [50, 58].

*Mimiviridae* comprised up to 45% of viral reads in Lake Levinson-Lessing and up to 14% in the other lakes, making it one of the most abundant families. Its abundance remained stable or declined with Holocene warming in Bolshoe Toko, Lama, Ulu, and Ilirney. In contrast, it increased more than sixfold in Lake Levinson-Lessing after 13.5 cal ka BP, becoming dominant alongside *Caudoviricetes*. *Mimiviridae*, the main representative of *Imitervirales* in lakes (median = 100%), is slightly less abundant in polar than temperate lakes [11], which may indicate a temperature-dependent distribution. Although this pattern could explain the Holocene increase, it contrasts with the higher abundance in Levinson-Lessing relative to warmer, lower-latitude lakes.

Lake Levinson-Lessing shared more viral taxa with marine cores than any other lake in the dataset (24 taxa; Supplementary Fig. S5), with higher abundances before 44.2 cal ka BP (Supplementary Fig. S15). This pattern is consistent with palaeogeological evidence that parts of the Taymyr Peninsula were covered by marine waters during this time [51]. Therefore, the viral community at the time likely reflected the more marine-like conditions. Such marine influence has not been documented for nearby lakes, including Lake Ilirney, highlighting the unique history of Lake Levinson-Lessing.

### Virus–host co-occurrence patterns on millennial timescales

**Figure 3:**
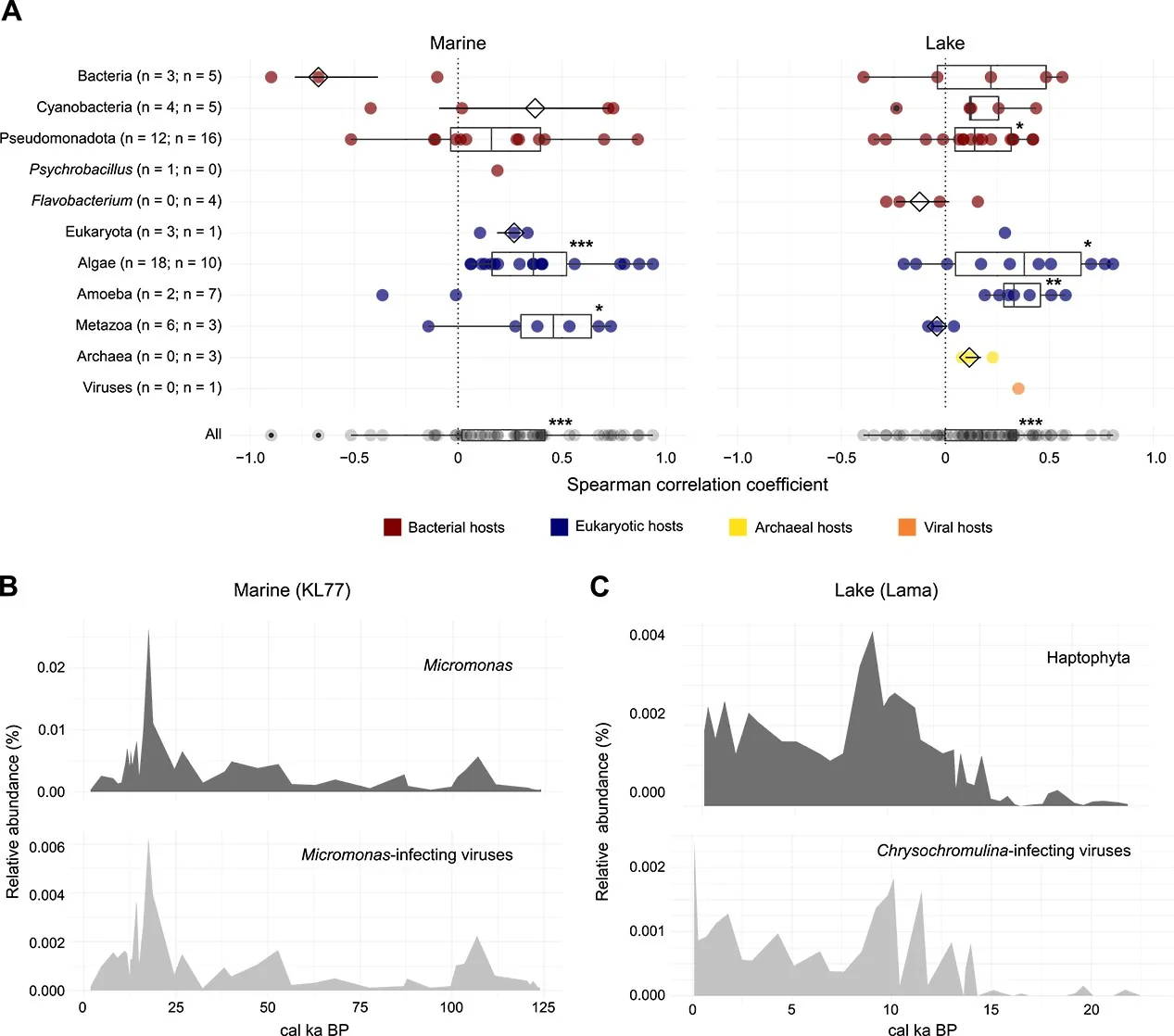
Abundance correlations of virus–host pairs. (A) Virus–host abundance correlations in marine (left) and lake (right) environments. Each point represents the Spearman correlation coefficient of a virus–host pair at a specific site. The number of correlations per environment is indicated. Host abundances were aggregated at the genus level for bacteria (red), the phylum level for eukaryotes (blue), and the superkingdom level for archaea (yellow). All correlations are summarised in a boxplot (black) at the bottom. Stars above boxplots indicate significance levels from one-sided Wilcoxon signed-rank tests [*P* ≤ 0.05 (^*^), *P ≤* .01 (^**^), *P ≤* .001 (^***^)]. (B) Temporal relative abundances of *Micromonas* (top) and *Micromonas*-associated viruses (bottom) at site KL77. (C) Temporal relative abundances of Haptophytes (top) and associated viruses (bottom) at Lake Lama. Relative abundances are shown as a proportion of total reads.

On millennial timescales, virus and host abundances were mostly positively correlated in both lake and marine environments (Fig. 3A). For this correlation analysis, we identified 83 virus–host pairs for the most abundant virus species, based on databases and peer-reviewed literature. These pairs involve 28 host taxa spanning all domains of life. This number is strongly constrained by current phage–host reference databases, which are particularly limited for freshwater systems compared to marine ones [94]. Thus, the true diversity of virus–host associations in these communities is likely greater than what is captured here. We calculated correlations using the cumulative relative abundance of viruses infecting the same host. Correlation coefficients were significantly greater than zero in both marine and lake systems (Wilcoxon signed-rank test, one-sided*, P <* .001). This is in line with our expectation that viruses must co-occur with their hosts on long-term scales, because sediment samples integrate occurrences of several decades. As the sampling resolution reflects variation on multi-millennial timescale, short-term virus–host dynamics cannot be resolved. The correlations observed here reflect long-term co-occurrence patterns between viruses and hosts rather than short-term infection dynamics. Because each sediment sample integrates DNA signals accumulated over several decades, the data capture broad ecological associations that persist through time, rather than transient infection or production cycles. Although virus–host interactions operate on short timescales, such short-term variability averages out in sedimentary DNA records, revealing only sustained relationships on ecological or millennial timescales.

Correlations were particularly strong between algal hosts and their viruses (Fig. 3A). For example, *Micromonas* and its viruses followed the same temporal patterns across 125 cal ka in core KL77 (Fig. 3B). In lakes such as Lama, we observed strong correlations between *Chrysochromulina*-infecting viruses and the broader Haptophyte phylum, which includes *Chrysochromulina* (Spearman = 0.81, *P <* .001; Fig. 3C). In aquatic systems, abundances of phytoplankton and their viruses are often tightly coupled, because they follow seasonal bursts and collapses during phytoplankton blooms [95]. Hence, strong correlations between them on longer timescales are expected.

### Introduction of Arctic-origin host strains and disrupted virus–host dynamics after the Bering Strait opening

**Figure 4:**
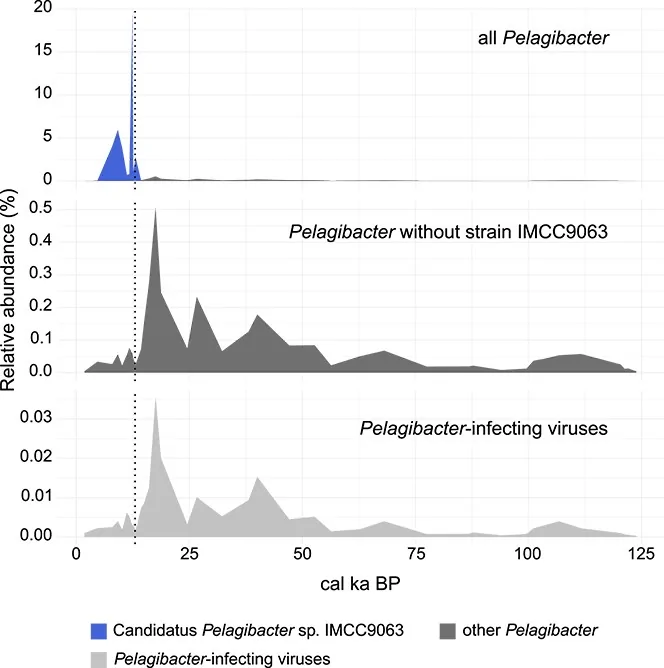
Temporal relative abundances of *Pelagibacter* and *Pelagibacter*-infecting viruses at site KL77. The upper plot shows all *Pelagibacter* species and strains, with strain IMCC9063 highlighted in blue and all others aggregated in dark grey. Due to the high relative abundance of IMCC9063, other *Pelagibacter* species are nearly undetectable in this plot. The middle plot displays *Pelagibacter* species excluding IMCC9063 (dark grey), revealing temporal dynamics of less dominant taxa. The lower plot shows *Pelagibacter*-infecting viruses (light grey). The dotted line indicates the timing of the Bering Strait opening.

Many abundant virus species from the North Pacific marine cores belong to *Pelagivirus*, which infects *Pelagibacterales* (SAR11), the most abundant bacterial clade in the oceans [96]. In KL77, *Pelagibacter*-infecting viruses were strongly correlated with their host in the Pleistocene (Spearman = 0.98*, P <* .001) but not in the Holocene (Spearman = 0.10*, P =* .785). This shift is driven by a single strain, Candidatus *Pelagibacter* sp. IMCC9063, which became highly abundant around 12.4 cal ka BP and sharply declined in abundance after 4.7 cal ka BP (Fig. 4). This strain was not correlated with any identified *Pelagibacter*-infecting virus in KL77 (Supplementary Fig. S16). Removing it restored the correlation between the remaining *Pelagibacter* species and their viruses, resulting in a strong positive Spearman coefficient of 0.86 (*P =* .004) also in the Holocene samples. A similar pattern was seen for *Synechococcus* (Supplementary Fig. S17). The strain *Synechococcus* sp. Ace-Pa dominated the genus around 12.4 cal ka BP and decreased in abundance shortly after. It skewed the correlation analysis suggesting a lack of correlation between virus and host (Spearman = −0.09, *P =* .811), but when excluded, the virus–host correlation became positive for the Holocene, although not statistically significant (Spearman = 0.48, *P =* .166). Another strain, *Synechococcus* sp. UW69, increased in abundance around 14.3 cal ka BP and disappeared after 11.2 cal ka BP. Although UW69 was less abundant (max = 0.07%) than strain Ace-Pa (max = 0.27%), its additional removal resulted in a statistically significant correlation between the remaining *Synechococcus* species and their known viruses (Spearman = 0.70, *P =* .031).

The abundant *Pelagibacter* and *Synechococcus* Ace-Pa strains are both associated with polar waters. Candidatus *Pelagibacter* sp. IMCC9063, isolated from the Arctic Ocean [97], is limited to waters <18.2°C [98] and carries cold-adaptation genes under selection in Arctic and Antarctic SAR11 strains [98]. *Synechococcus* sp. Ace-Pa, isolated from a marine Antarctic Lake [99], has not yet been found elsewhere, though a related strain may also occur in the Arctic. The *Synechococcus* strain UW69 is not found in the Arctic Ocean in modern samples but is associated with transitional waters between cold, nutrient-rich waters and warm, oligotrophic waters [100].

Despite favourable cold-period conditions, both strains became abundant only after the Bering Strait opened (~11–12 ka BP) [18, 19]. This suggests their introduction from the Arctic Ocean into the Bering Sea following the initial flooding of Beringia. The increase in abundance of *Synechococcus* strain UW69 prior to the opening of the Bering Strait coincided with intense meltwater pulses into the ocean and may be an indicator of the changing environmental conditions in the Bering Sea at the time [22, 23]. No identified *Pelagibacter*-infecting virus mirrored the host peak at this time, indicating the absence of viral regulation, or infection by specific viruses not represented in current genome databases. Although the absence of a respective virus species in this dataset does not imply a general lack of viral infection of these strains at the time, their strong dominance in the bacterial community supports the hypothesis that extensive viral lysis did not occur. Their decrease in relative abundance after 9 cal ka BP may be explained by environmental changes such as increasing sea surface temperatures [40, 44] which became less suitable for cold-adapted *Pelagibacter* and *Synechococcus* species.

### Mismatch between bacteriophages and bacterial host class composition

**Figure 5:**
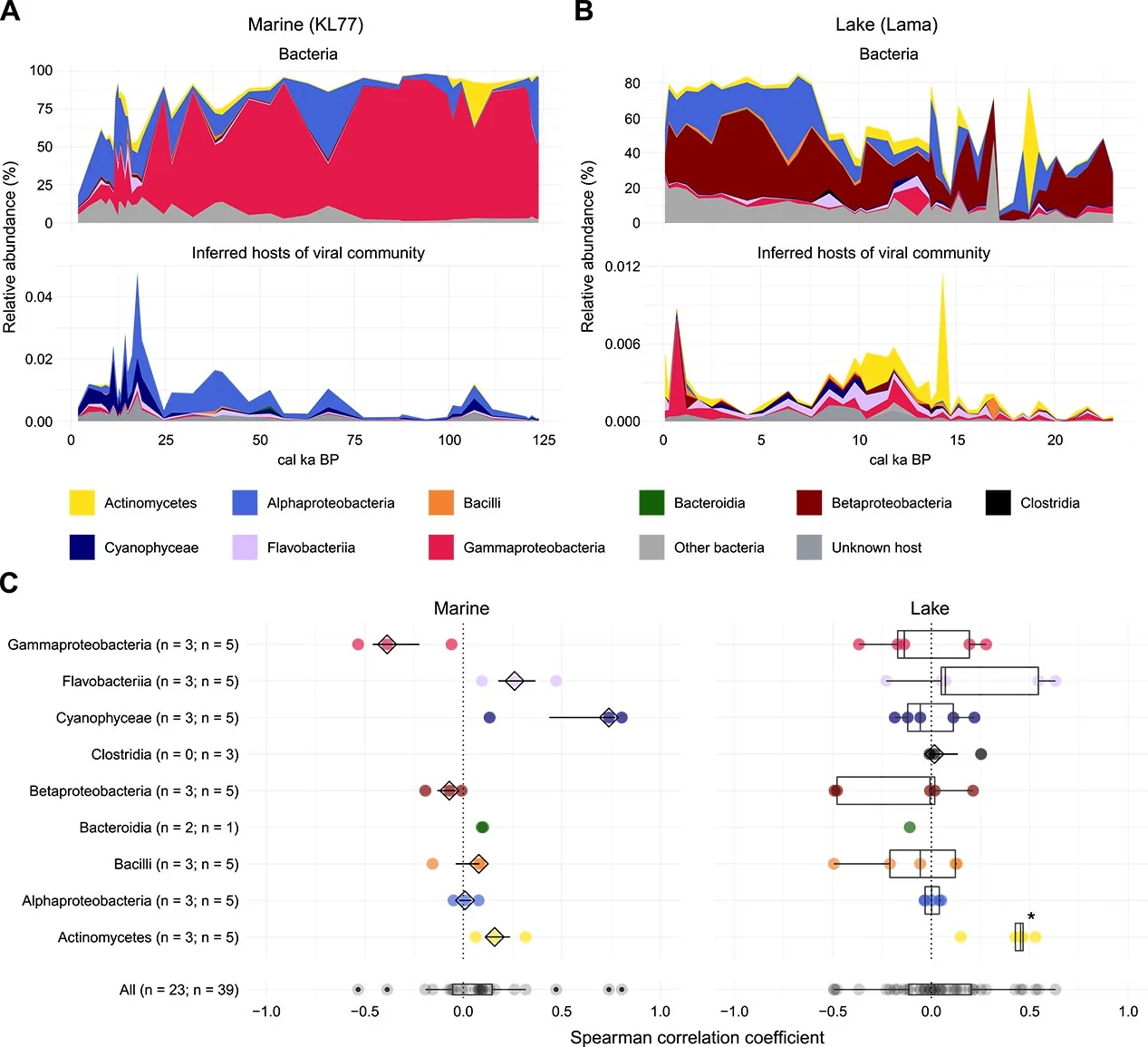
Ancient bacteria and their associated bacteriophages. (A) Temporal relative abundances of bacterial classes (top) and their associated bacteriophages (bottom) in cores KL77 and (B) Lake Lama. The colours indicate the bacterial classes and the inferred hosts of the associated viruses. (C) Boxplots of Spearman correlation coefficients between bacterial classes and associated bacteriophages in marine (left) and lake (right) environments. Each point represents the correlation at a specific site. Samples are coloured according to bacterial class (as in A and B). Combined distributions of all correlations are shown in black at the bottom of each panel. Stars above boxplots indicate significance levels from one-sided Wilcoxon signed-rank tests [*P ≤* .05 (^*^), *P ≤* .01 (^**^), *P ≤* .001 (^***^)].

The most abundant bacteriophages did not match the dominant bacterial classes in marine or lake systems (Fig. 5A and B). Pleistocene marine communities were dominated by *Gammaproteobacteria* (Fig. 5A), in line with contemporary marine environments [101], but their viruses were rare. However, phage communities in marine systems were dominated by cyanobacteria-infecting viruses, whereas their hosts, *Cyanophyceae*, were rare (median = 0.10%) (Fig. 5A). *Alphaproteobacteria* were the only group to co-occur with abundant viruses in both marine cores KL77 and KL12 during the Holocene. In lakes, *Betaproteobacteria* and *Alphaproteobacteria* occurred as dominant bacterial groups (Fig. 5B), consistent with modern observations [102, 103]. Corresponding phage communities were dominated by viruses infecting *Actinomycetes*, *Flavobacteria*, and *Gammaproteobacteria*.

Only *Actinomycetes* in lakes showed a significant positive correlation with their viruses (Fig. 5C), indicating sustained co-occurrence over millennial timescales. This pattern was particularly evident in Lake Ulu, where both *Actinomycetes* and their viruses remained highly abundant throughout the Pleistocene and Holocene (Supplementary Fig. S18). Furthermore, despite the low relative abundance of *Cyanophyceae*, they correlated strongly with their viruses in the marine system but not in the lakes (Fig. 5C). All other highly abundant bacterial classes and their viruses did not correlate in either lake or marine environments. In Lake Lama and Lake Ulu, *Cyanophyceae* and their viruses even showed antagonistic patterns (Supplementary Fig. S19 and S20), reflected by negative correlation coefficients (Fig. 5C). These antagonistic patterns can also be observed between the whole bacterial and bacteriophage communities at Lake Ilirney (Supplementary Fig. S21) as well as in marine cores KL12 (Supplementary Fig. S22) and KL77 (Fig. 5A). Additional negative correlations include *Pseudomonas* in Lake Ilirney (Supplementary Fig. S23), *Gammaproteobacteria* in lakes Bolshoe Toko, Ilirney and Levinson-Lessing and subarctic marine cores KL12 and KL77 (Supplementary Figs S24–S28) and *Betaproteobacteria* in lakes Bolshoe Toko and Ilirney (Supplementary Fig. S29 and S30).

Although short-term antagonism fits the Kill-the-Winner model [95], long-term data should show covariation due to cumulative signals. Instead, these antagonistic patterns on millennial timescales may result from other factors. However, during periods of high virus abundance, their host abundance is not zero, indicating that the host was present but at low relative abundances. Ongoing viral infection and lysis of a host group allows organisms to succeed in the competition for growth resources, which shifts the composition in favour of the organisms that experience less pressure from viruses [104]. Therefore, an increase in abundance of a virus can coincide with host declines due to competition. Conversely, reduced viral pressure can allow host populations to rebound. These shifts may reflect co-evolutionary dynamics described by the Red Queen hypothesis, although they likely occur on much shorter time-scales [9]. However, antagonism may also reflect viral shifts between lysogenic and lytic cycles [4, 105, 106]. Lysogenic viruses produce little viral DNA [107], making them harder to detect in sedaDNA datasets. Integrated viruses may even benefit hosts relative to competitors [104]. Yet, when the viruses revert to a lytic lifestyle, the host can no longer establish dominance within their community, and they decline in relative abundance. This phenomenon of suppressed host dominance through viral lysis may explain both antagonistic temporal patterns and mismatches between dominant bacteriophages and bacteria. Furthermore, environmental factors may also influence virus–host encounter rates and thereby modulate infection dynamics. For example, lower temperatures or reduced UV exposure, such as during periods with frozen lake surfaces, can stabilize virions and prolong their persistence in the environment [108]. Conversely, strong glacial meltwater input can introduce sediment particles that bind virions, reducing their availability for infection [109]. However, these interpretations must be viewed with caution, as current viral databases, particularly for freshwater phages, remain sparse and biased [94]. As reference collections expand, such relationships should be re-evaluated to refine interpretations of long-term virus–host dynamics.

In contrast to the general mismatches between bacterial and viral abundances, *Actinomycetes* in lakes showed a distinct pattern of sustained virus–host coupling (Fig. 5C). Freshwater *Actinobacteria* are among the most abundant and ubiquitous members of lake bacterioplankton [103], and their viruses have been described as one of the most diverse, abundant, and widespread viral groups in freshwater systems, with evidence for continuous replication and host lysis in lake water columns [110]. A comparable pattern can be seen for *Alphaproteobacteria* in marine environments, dominated by the SAR11 clade including the *Pelagibacter* genus. The success and ubiquitous abundance of the SAR11 clade was previously attributed to viral resistance [2]. However, this theory was later disproved due to the high abundance of pelagiphages [96]. The high relative percentage of pelagiphages throughout the late Pleistocene and Holocene implies sustained virus production, presumably reliant on host lysis, and thus challenges the viral-resistance hypothesis. In both systems, abundant host lineages and their viruses suggest stable long-term co-occurrence under persistent viral pressure [111].

Virus–host abundance patterns over millennial timescales show that virus–host correlations are not consistently positive, and that decoupled or antagonistic patterns may reflect ecologically meaningful processes. It is therefore important to consider that negative correlations between viruses and their hosts do not necessarily indicate false associations, but may reflect shifts in infection dynamics, particularly across environmental transitions such as glacial–interglacial warming. Such dynamics may be more common over long timescales than previously assumed and should be accounted for when using co-occurrence-based approaches to infer host-virus relationships in ancient datasets.

## Conclusions

This study demonstrates the feasibility of using sedaDNA time-series to reconstruct ancient DNA virus communities in both lake and marine environments, providing insights into long-term virus–host dynamics across geological epochs. Our results show that temporal patterns in ancient viromes are preserved and biologically meaningful, despite low proportions of viral reads.

By comparing lake and marine viromes, we find that distinct viral community signatures persist over millennial timescales and through major environmental changes. This persistent separation underscores the importance of ecosystem-specific processes in shaping viral assemblages. Whereas marine viral communities are more interconnected, likely due to ocean circulation, lake communities are more isolated and show high levels of endemism, including a marine-like viral signature in Lake Levinson-Lessing, likely reflecting submergence by marine waters at that time. These findings align with contemporary patterns and extend them back through glacial–interglacial transitions.

Viral community composition differs between glacial and interglacial periods, with environment (lake vs marine) explaining a small but significant amount of the virus community composition. Lakes also have greater inter-site dissimilarity than marine systems, supporting the idea that local conditions play a dominant role in shaping freshwater viral communities. Temporal structure reveals clear ecological responses to climatic shifts. Lakes show shifts in dominant viral taxa at the onset of the Holocene, including increases in algal viruses with rising aquatic productivity, and the disappearance of cold-associated phage families like *Casjensviridae*. In marine systems, transitions such as the Antarctic Cold Reversal and the postglacial opening of the Bering Strait coincide with marked shifts in virus–host associations. These patterns suggest that ancient viral communities tracked both local and global environmental changes across time.

Using a literature-based approach, we identified 83 virus–host pairs and found that most exhibit positive correlations in their relative abundances, consistent with long-term co-occurrence. These correlations are particularly strong for phytoplankton and their viruses, reflecting tight ecological coupling. However, we also detect non-correlated and antagonistic patterns, some of which we could link to geological events (e.g. Bering Strait opening) and hypothesised life-cycle switches between lytic and lysogenic phases or long-term changes in contact rates in response to environmental change. Our results highlight the importance of considering negative correlations between viruses and their hosts when applying co-occurrence-based approaches to infer virus–host relationships. Such antagonistic patterns may be common over long timescales, especially across warm–cold transitions or other major environmental changes that affect infection dynamics.

Despite promising insights into long-term viral community structure and virus–host patterns derived from sedaDNA time series, several challenges remain. Interpretation of ancient viromes is constrained by potential taphonomic and database biases, although the specific mechanisms driving preservation or underrepresentation of particular viral groups, such as those differing greatly in virion size or structure, are not yet understood. Physical properties such as virion size, capsid stability, surface charge, and hydrophobicity can influence the binding of viruses to sediment particles [112–114] and thereby affect their likelihood of preservation, but such effects cannot currently be disentangled from ecological drivers such as host abundance and virus production [109, 115]. These factors, together with a high proportion of unassigned reads and the limited coverage of current viral reference databases, restrict the resolution of sedaDNA-based virome reconstructions. Future studies should expand reference collections, pursue genome-resolved reconstructions, and aim to integrate RNA viruses, ultimately providing a deeper understanding of how viral–host interactions evolve over long timescales and respond to environmental change.

## Supplementary Material

SupplementaryInformation_wrag025

SupplementaryTable1_wrag025

SupplementaryTable2_wrag025

SupplementaryTable3_wrag025

## Data Availability

This study uses sedaDNA shotgun datasets previously published [49]. The respective raw shotgun datasets from four lake sediment cores are available under the BioProject accessions PRJEB94536 (Lake Levinson-Lessing), PRJEB80635 (Lake Ilirney), PRJEB80642 (Lake Bolshoe Toko) and PRJEB82635 (Lake Ulu) at the European Nucleotide Archive (ENA). Additional sedaDNA shotgun data from Lake Lama (PRJEB80877) [54] and from marine sediment cores PS97/72–01 (PRJEB74305) [47], SO201–2-77KL (PRJEB66300) [40] and SO201–2-12KL (PRJEB66300) [68] were included in this study. The documentation of the bioinformatic workflows, metadata, taxonomic reference indexing resources of the taxonomic database and an example script for filtering the final datasets is publicly available [49]. Code and data required to reproduce the figures are publicly available at Zenodo (https://zenodo.org/records/18429384).
