# Supplementary material for: DNA virus–host patterns in lake and marine environments over the last glacial cycle
Source: ISME J. 2026 Feb 19;20(1):wrag025. doi: 10.1093/ismejo/wrag025 (PMC13037465; doi:10.1093/ismejo/wrag025)
Supplement: SupplementaryInformation_wrag025 [file supplementaryinformation_wrag025.pdf]

Supplementary Information

Supplementary Table 1
Comma-separated file containing sample metadata including site, core, sample, age (years), age (ka), number of reads, number of mapped reads, percent mapped reads, latitude, longitude, age–depth model DOI, and ENA Project ID.

Supplementary Table 2
Comma-separated file containing contamination taxa with total read counts (# Total Reads), read counts in blanks (# Reads in Blanks), and the percentage of reads in blanks relative to all reads in blanks and samples (% Reads in Blanks).

Supplementary Table 3
Tab-separated file containing virus–host pairings, including virus name (taxon), optional alternative name (alternative name), assigned host (host), and the reference or database used for host assignment (reference). The table also includes the corresponding host taxon in the dataset (host_taxon), the host taxon used for correlation analysis (host_taxon_correlation), the assigned host group (host_group), and host superkingdom (host_superkingdom).

Supplementary Figures
Supplementary Figures 1 and 2. Ancient pattern results of viruses and bacteria.
Supplementary Figure 3. Rarefaction analysis results.
Supplementary Figure 4. Relative abundance patterns of uncultured Caudovirales phage.
Supplementary Figures 5 and 6. Results of the sediment core similarity analysis.
Supplementary Figures 7–14. Relative abundances of abundant virus taxa in individual sediment cores.
Supplementary Figure 15. Relative abundance of viruses shared between Lake Levinson-Lessing with the marine cores.
Supplementary Figure 16. Correlation of *Pelagibacter*-infecting viruses with the *Pelagibacter* strain IMCC9063.
Supplementary Figure 17. Relative abundances of *Synechococcus* and *Synechococcus*-infecting viruses in core KL77.
Supplementary Figure 18. Relative abundances of bacterial classes and their corresponding viruses in Lake Ulu.
Supplementary Figures 19–30. Relative abundances of viruses and their hosts showing antagonistic patterns.

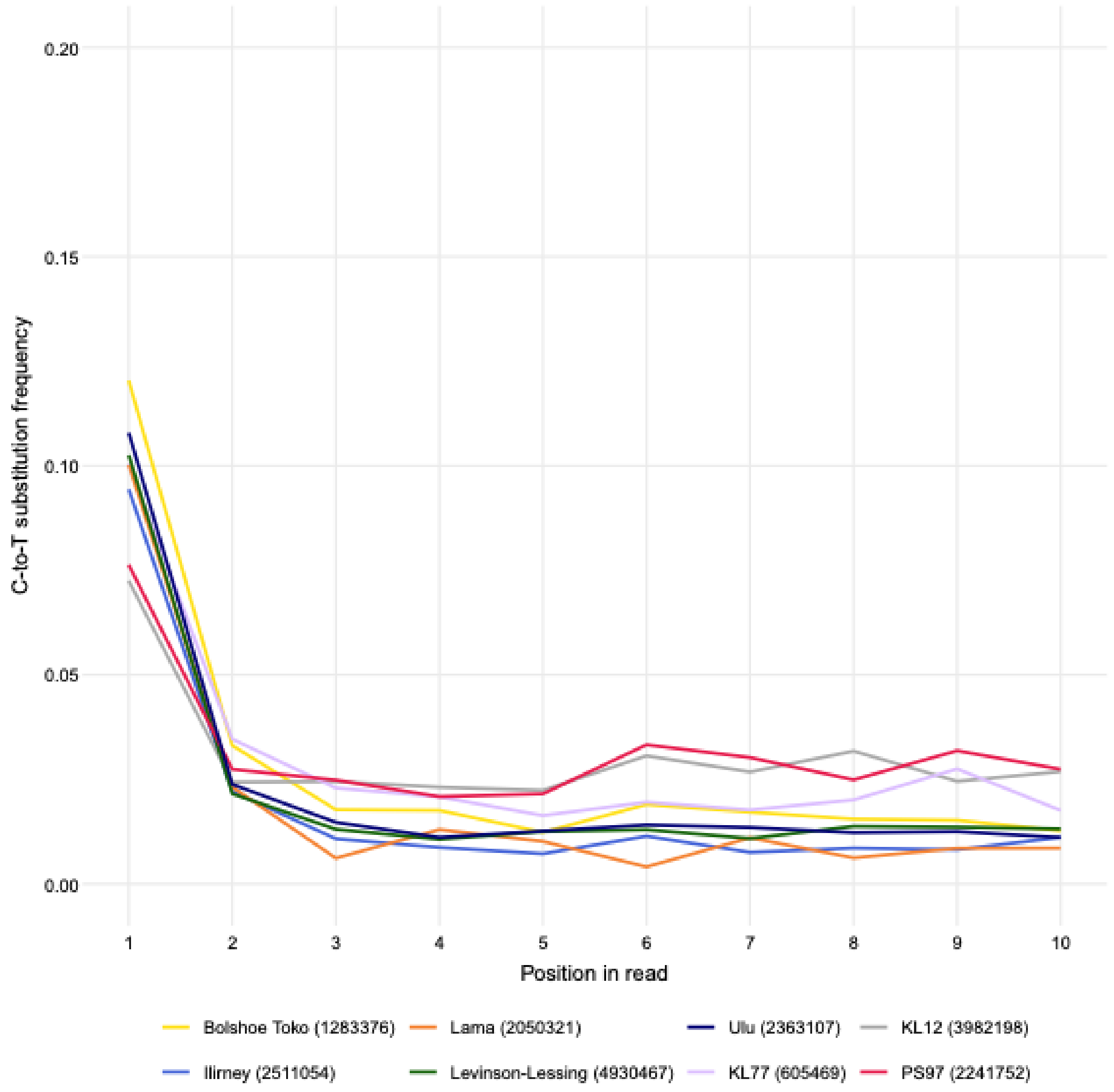

SFig. 1: Post-mortem damage patterns of viruses by read position. The line shows mean values of the individual cores, which are indicated by colour. The C-to-T substitution frequencies are shown for contigs with a prediction accuracy ≥0.6 and length ≥1000 bp. The number of reads aligned to these contigs per lake is shown in brackets.

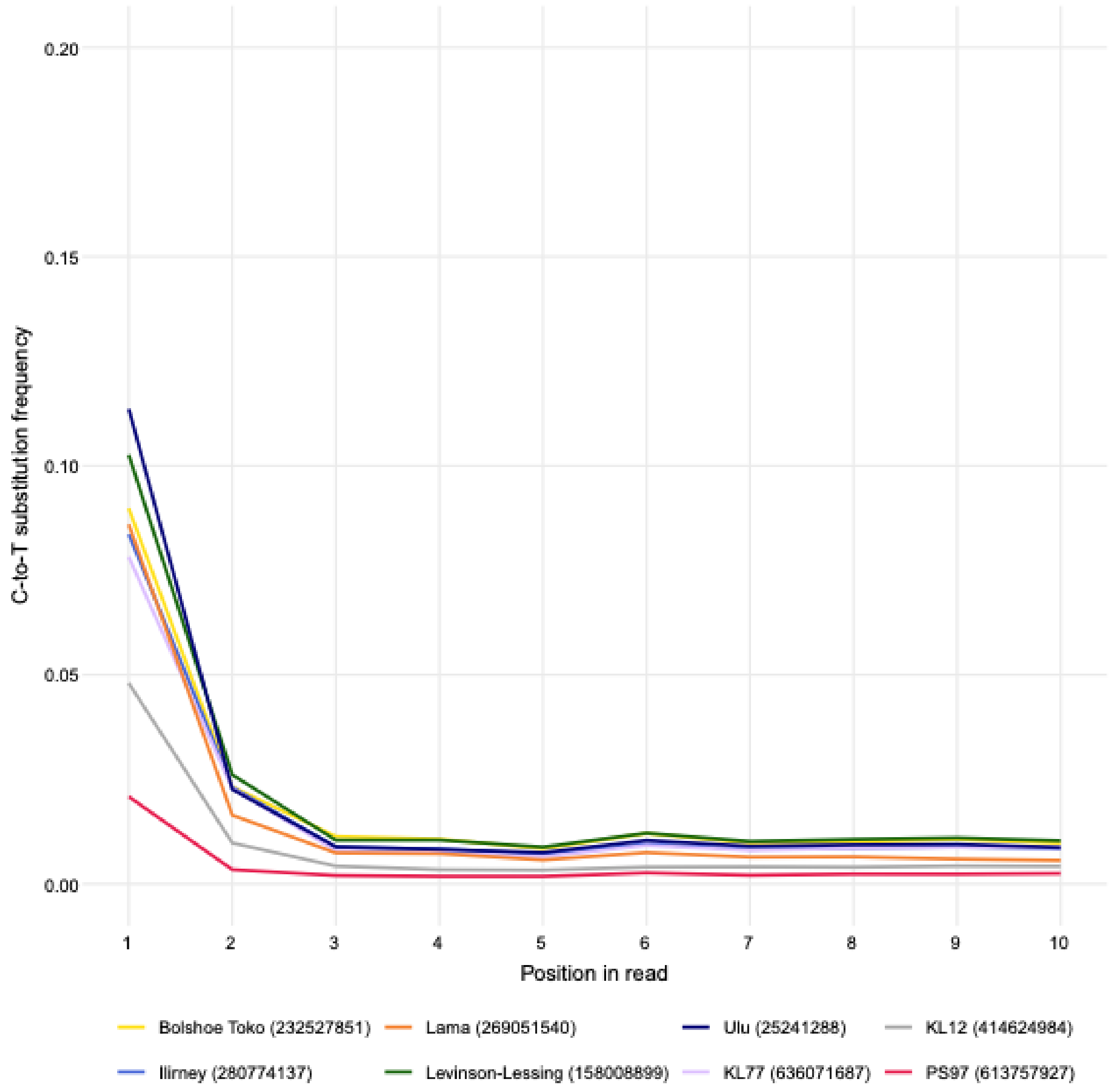

SFig. 2: Post-mortem damage patterns of bacteria by read position. The line shows mean values of the individual cores, which are indicated by colour. The C-to-T substitution frequencies are shown for contigs with a prediction accuracy ≥0.6 and length ≥1000 bp. The number of reads aligned to these contigs per lake is shown in brackets.

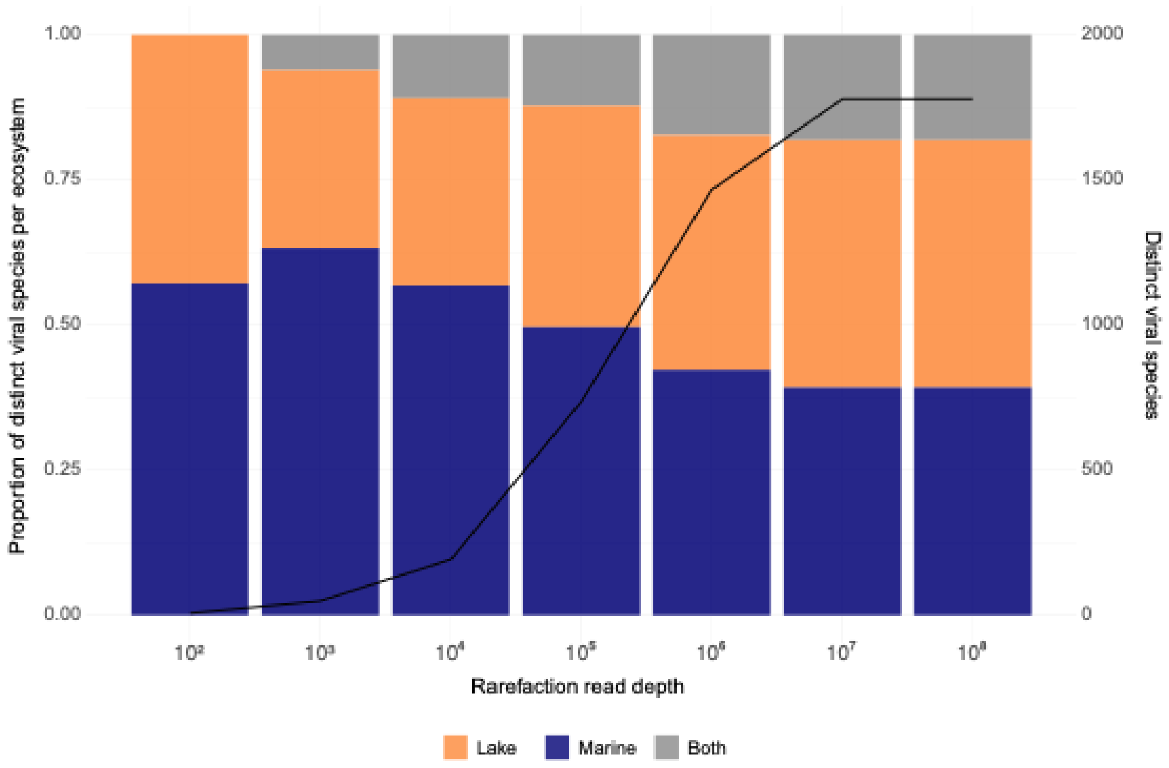

SFig. 3: Effect of rarefaction depth on the distribution of viral taxa between environments. Stacked bar plots show the proportion of viral species detected uniquely in lakes (orange), uniquely in marine cores (blue), or shared between both environments (grey) across rarefaction depths ranging from 10^2 to 10^8 reads. The black line indicates the total number of distinct viral species recovered at each read depth (right axis).

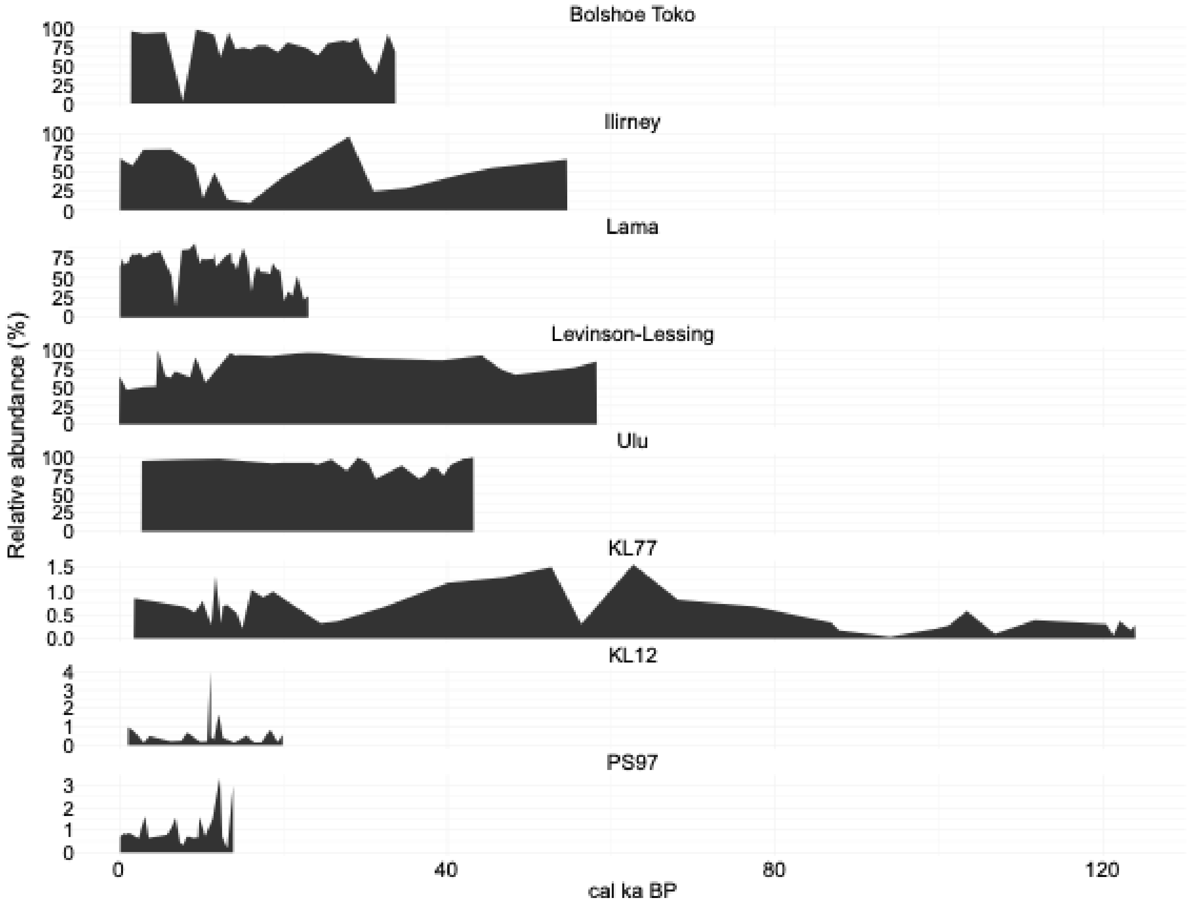

SFig. 4: Relative abundance of uncultured Caudovirales phage in terrestrial cores. The relative abundance is shown in respect to all viral reads.

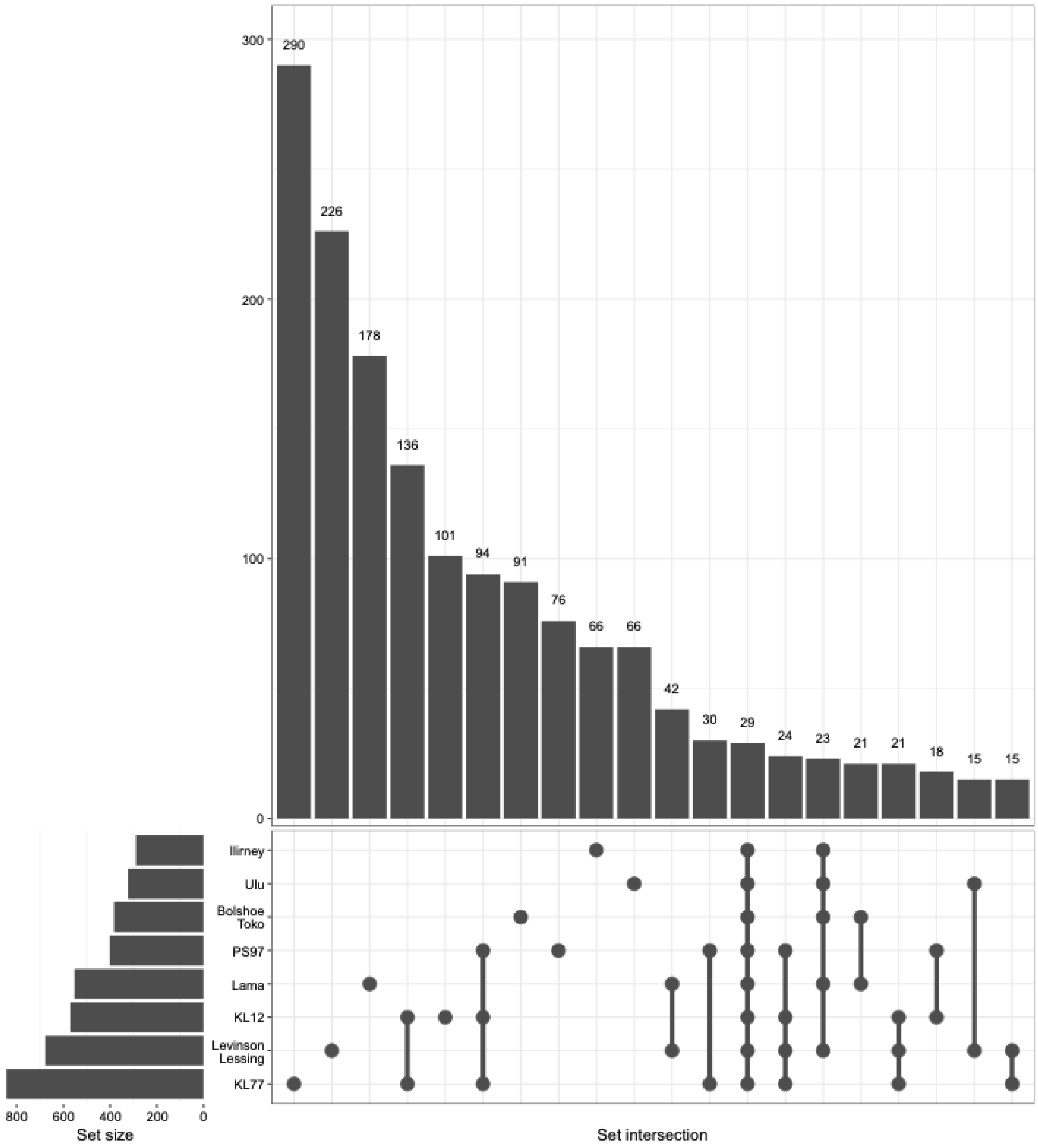

SFig. 5: Upset plot of the virus species. The number of viral species is shown on the left and shared taxa with other sites are indicated in the upper plot.

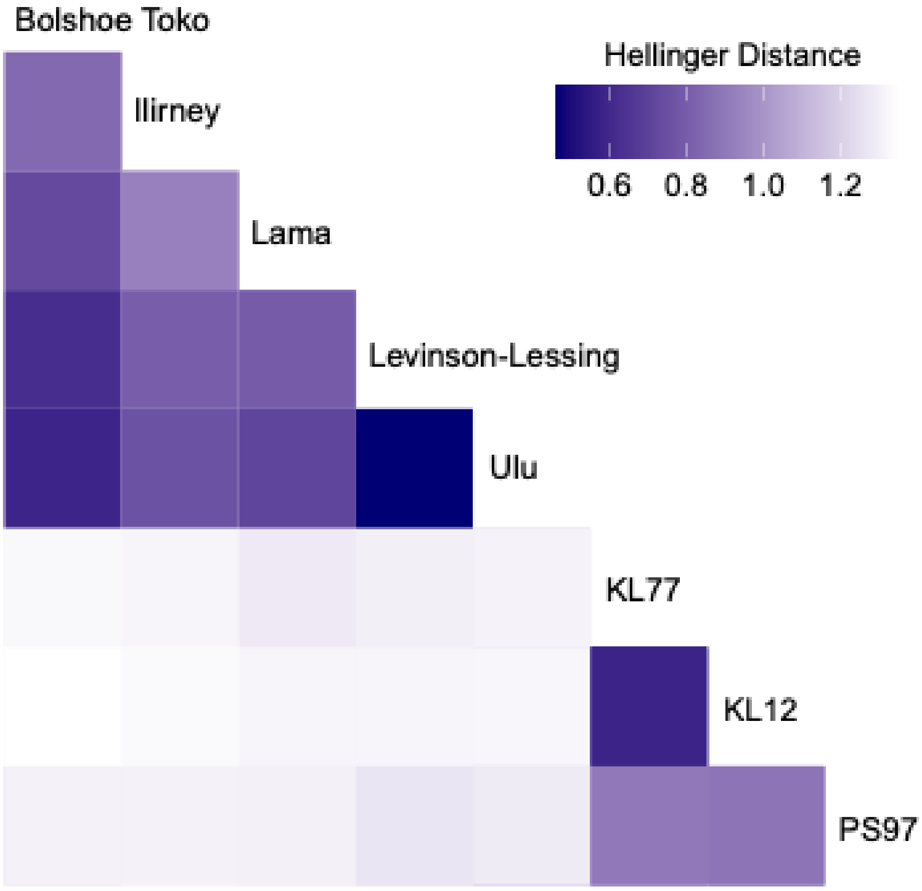

SFig 6: Heatmap of pairwise Hellinger distances of the virus species community between sites. Darker shading indicates higher similarity.

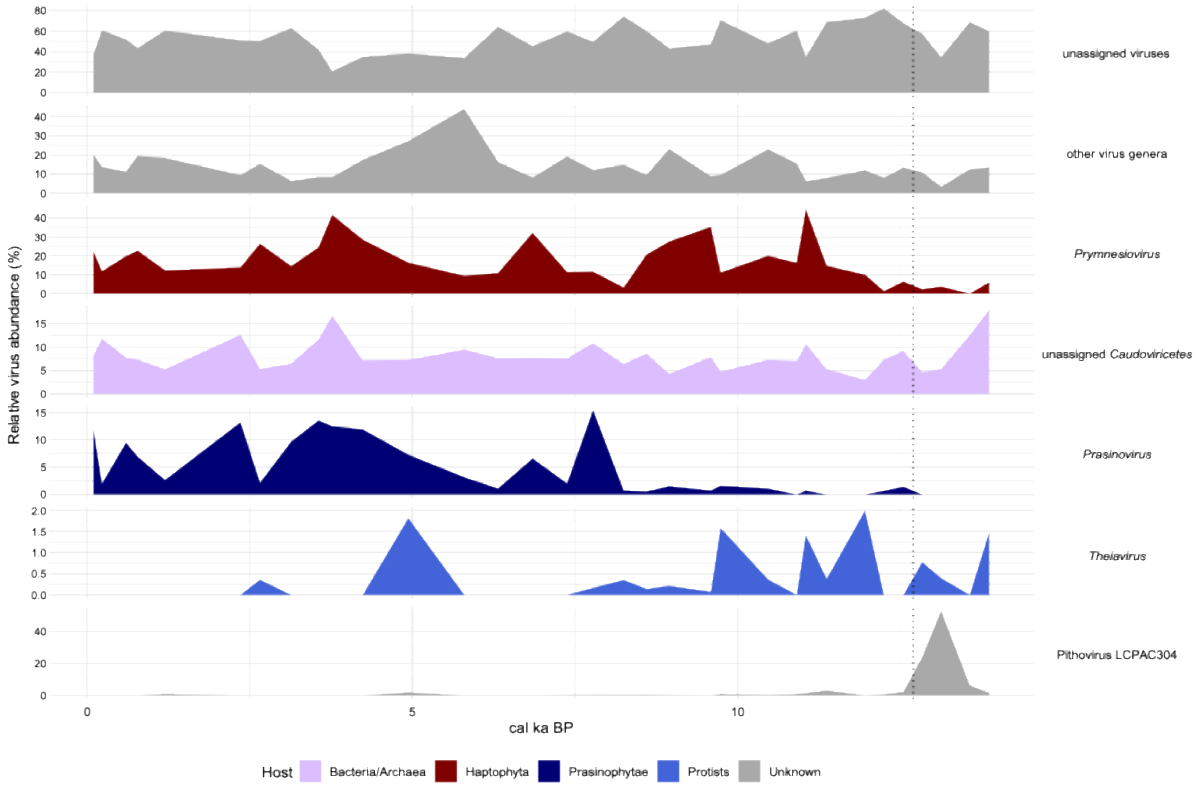

SFig 7: Temporal patterns of abundant virus genera and the Pithovirus strain LCPA304 in PS97. The relative abundance is shown in respect to all viral reads. Colors indicate the inferred host group. The dashed line marks the end of the ACR (~12.7 ka BP).

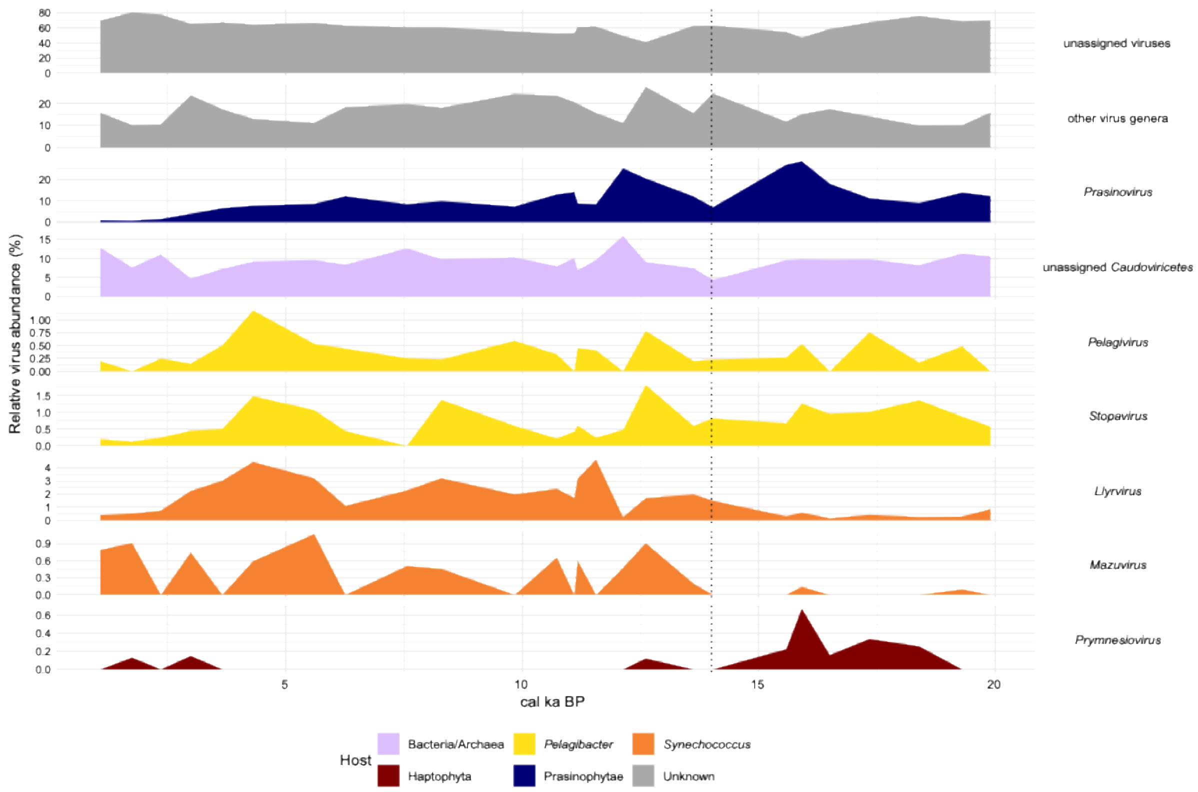

SFig 8: Temporal patterns of abundant virus genera in KL12. The relative abundance is shown in respect to all viral reads. Colors indicate the inferred host group. The dashed line marks the beginning of global warming at 14 ka BP.

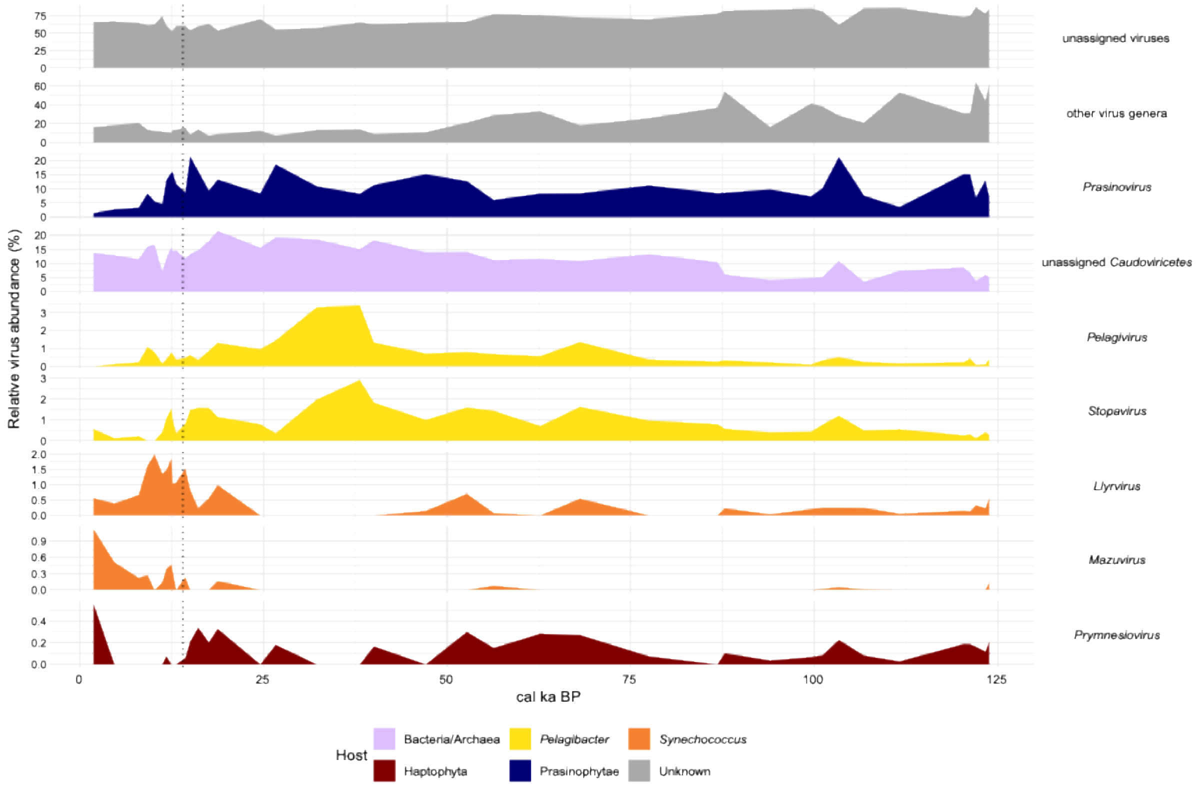

SFig 9: Temporal patterns of abundant virus genera in KL77. The relative abundance is shown in respect to all viral reads. Colors indicate the inferred host group. The dashed line marks the beginning of global warming at 14 ka BP.

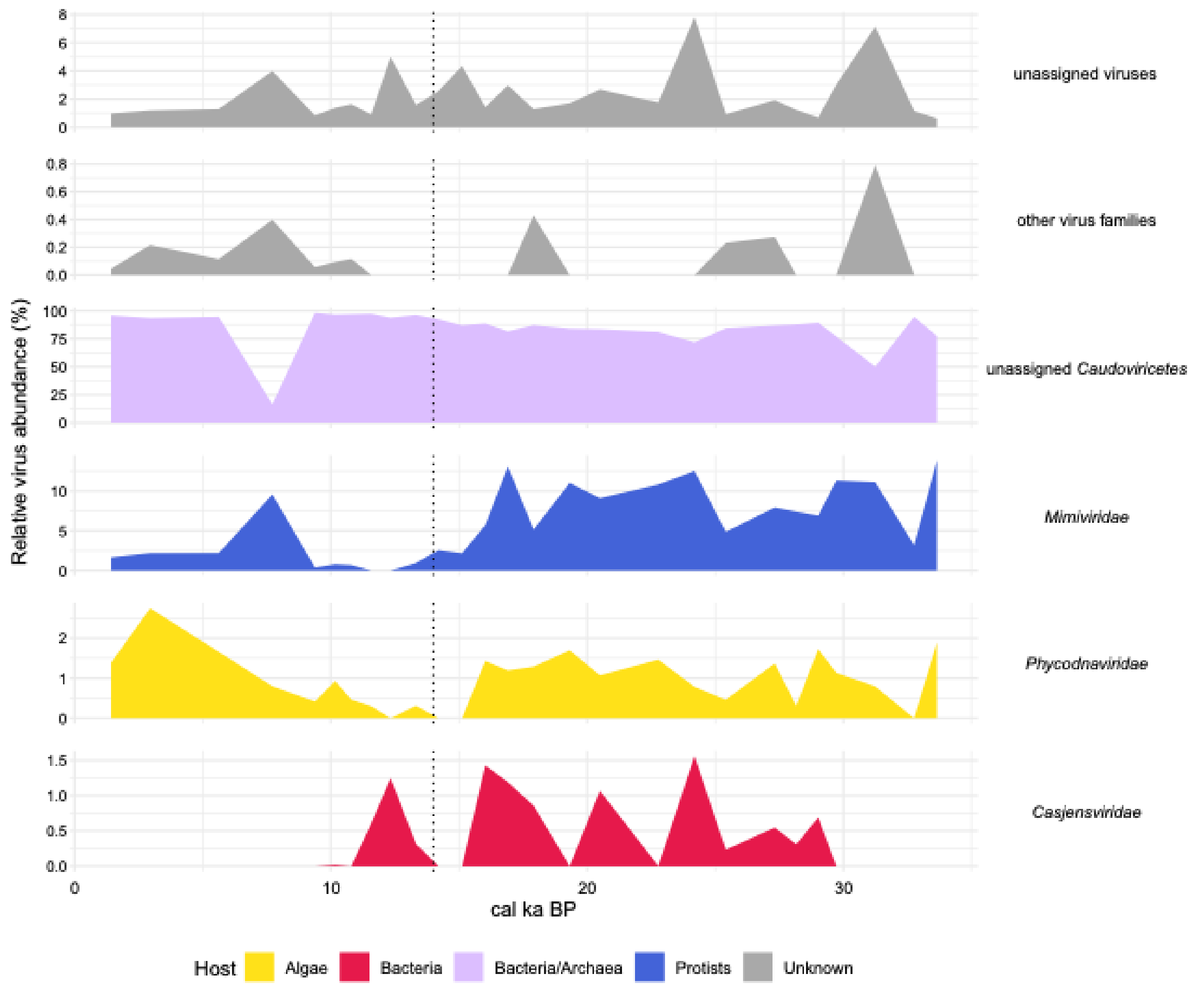

SFig. 10: Temporal patterns of abundant virus families in Lake Bolshoe Toko. The relative abundance is shown in respect to all viral reads. Colors indicate the inferred host group. The dashed line marks the beginning of global warming at 14 ka BP.

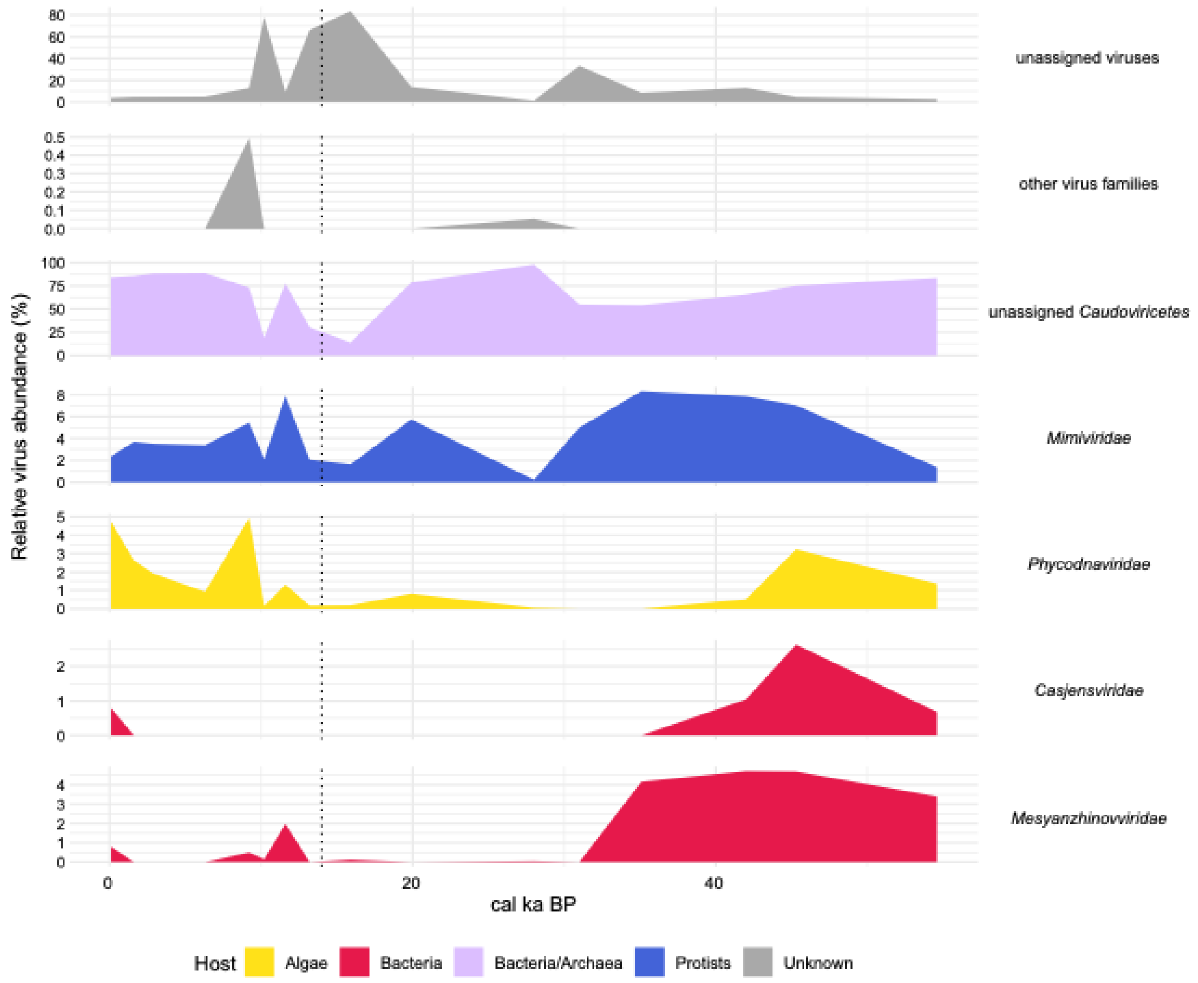

SFig. 11: Temporal patterns of abundant virus families in Lake Ilirney. The relative abundance is shown in respect to all viral reads. Colors indicate the inferred host group. The dashed line marks the beginning of global warming at 14 ka BP.

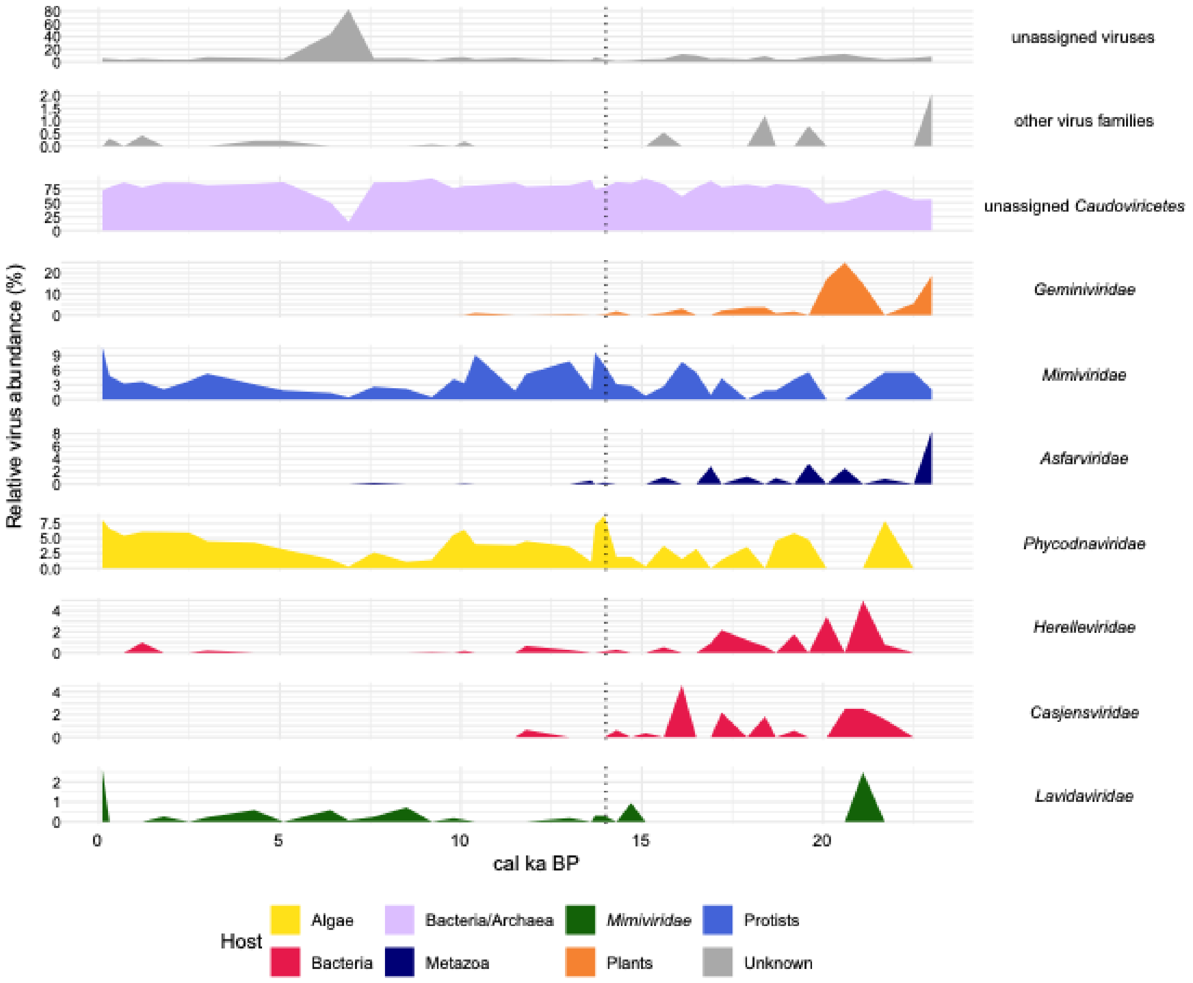

SFig. 12: Temporal patterns of abundant virus families in Lake Lama. The relative abundance is shown in respect to all viral reads. Colors indicate the inferred host group. The dashed line marks the beginning of global warming at 14 ka BP.

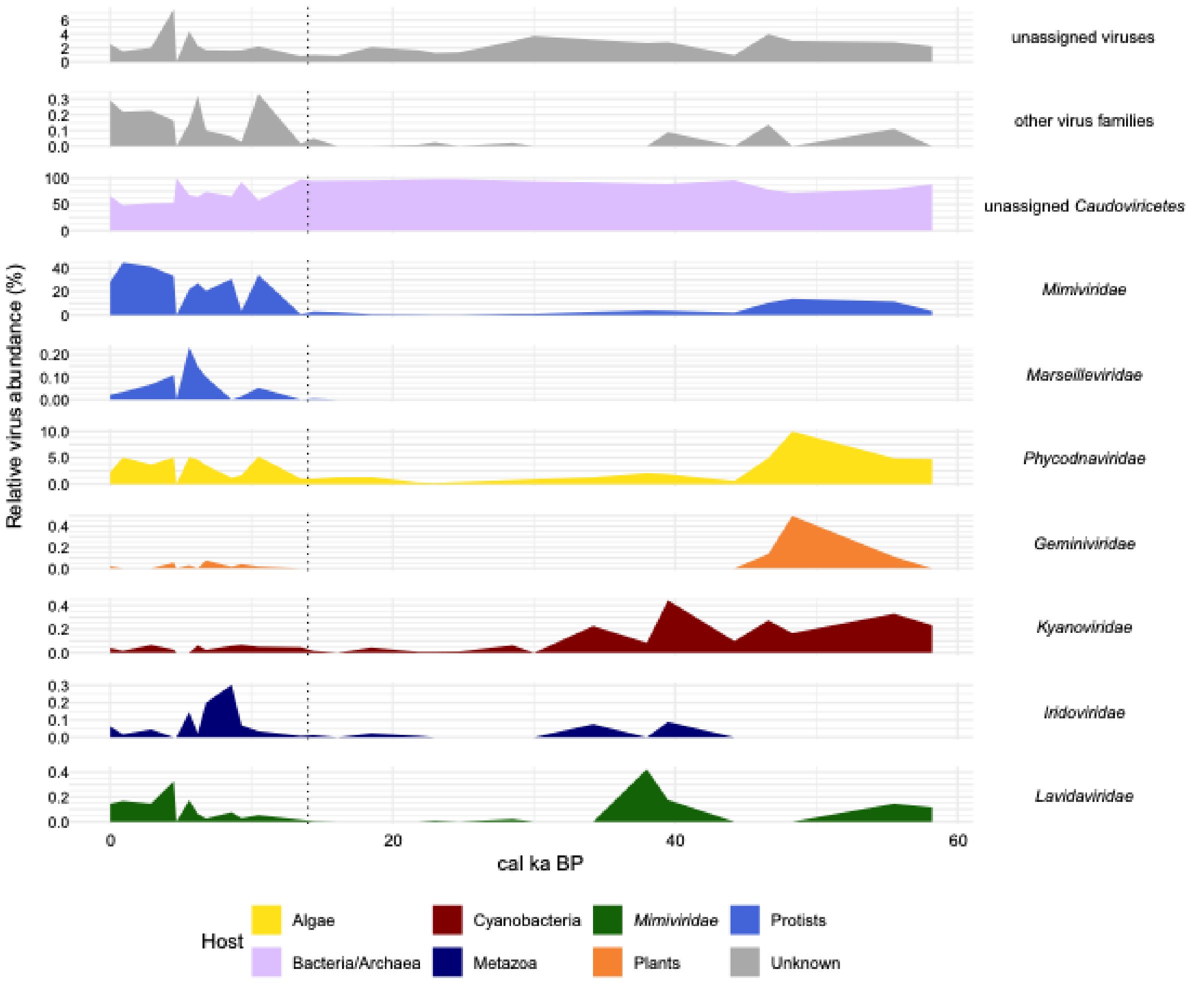

SFig. 13: Temporal patterns of abundant virus families in Lake Levinson-Lessing. The relative abundance is shown in respect to all viral reads. Colors indicate the inferred host group. The dashed line marks the beginning of global warming at 14 ka BP.

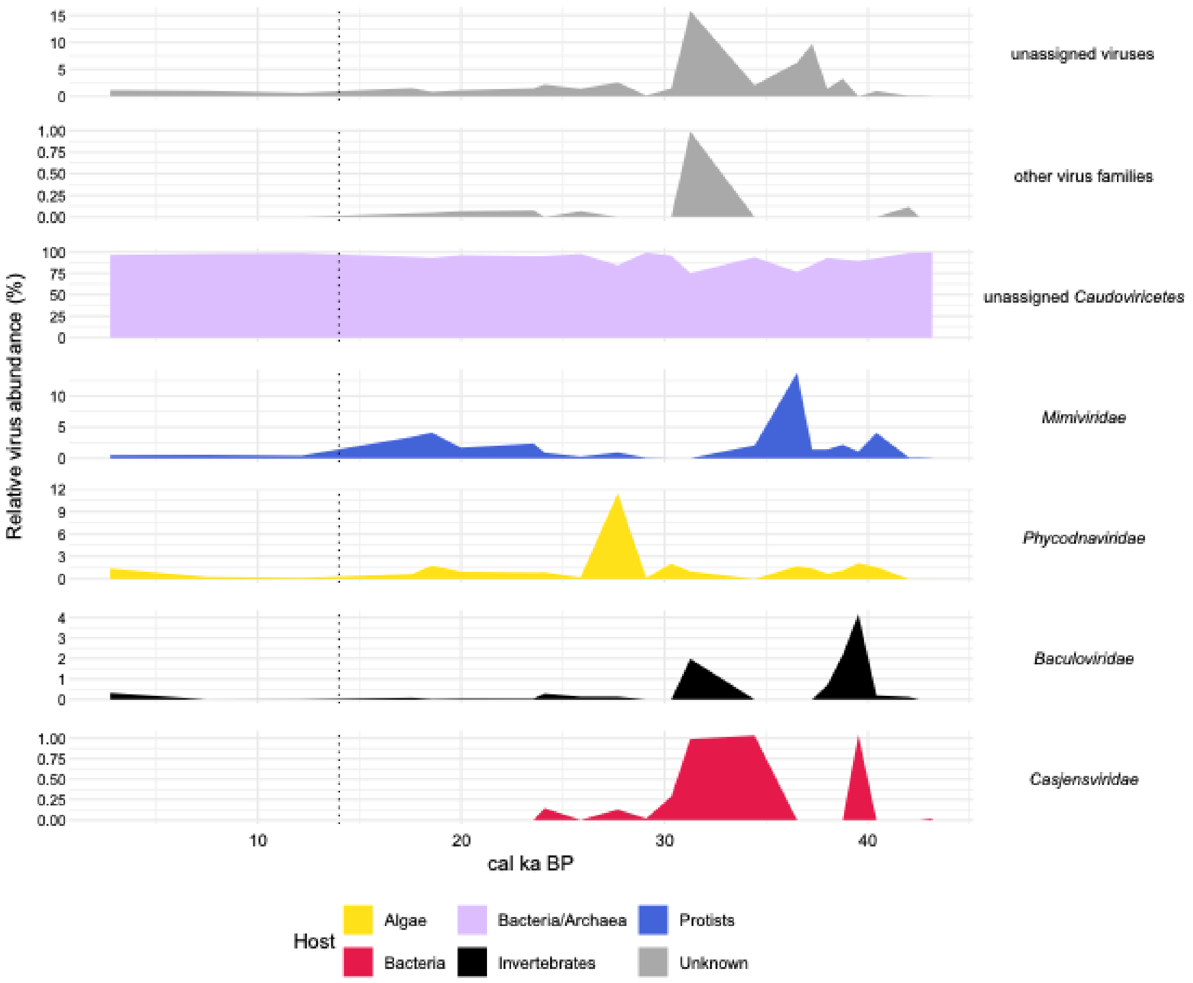

SFig. 14: Temporal patterns of abundant virus families in Lake Ulu. The relative abundance is shown in respect to all viral reads. Colors indicate the inferred host group. The dashed line marks the beginning of global warming at 14 ka BP.

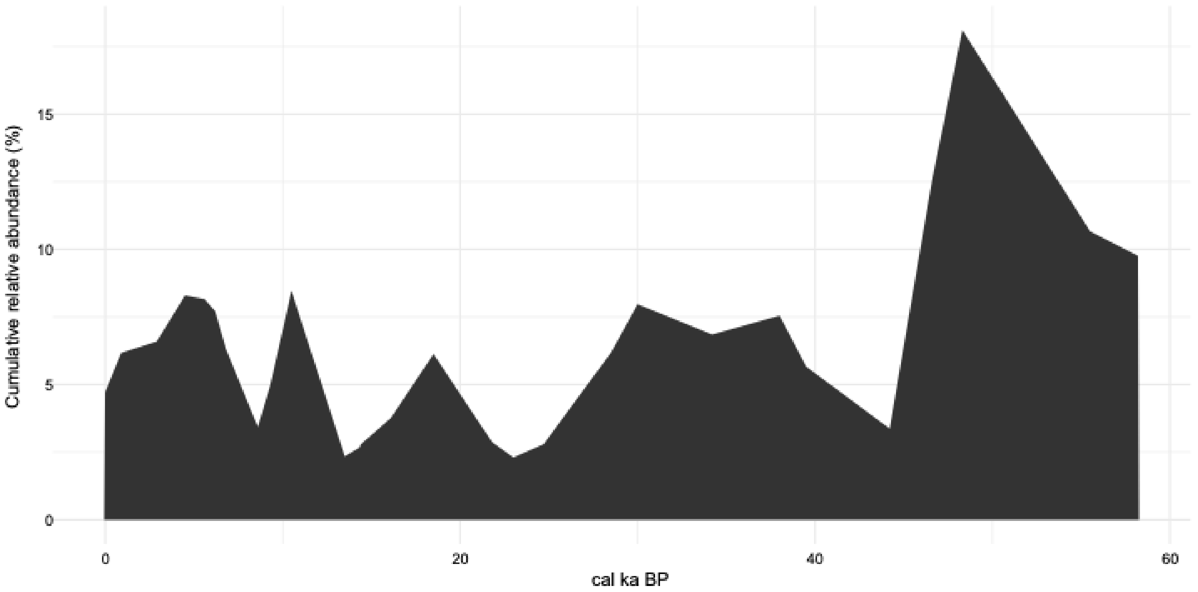

SFig. 15: Cumulative abundance of viruses that are shared between marine and any Lakes (without uncultured Caudovirales phage) in Lake Levinson-Lessing. The cumulative relative abundance is shown in respect to all viral reads.

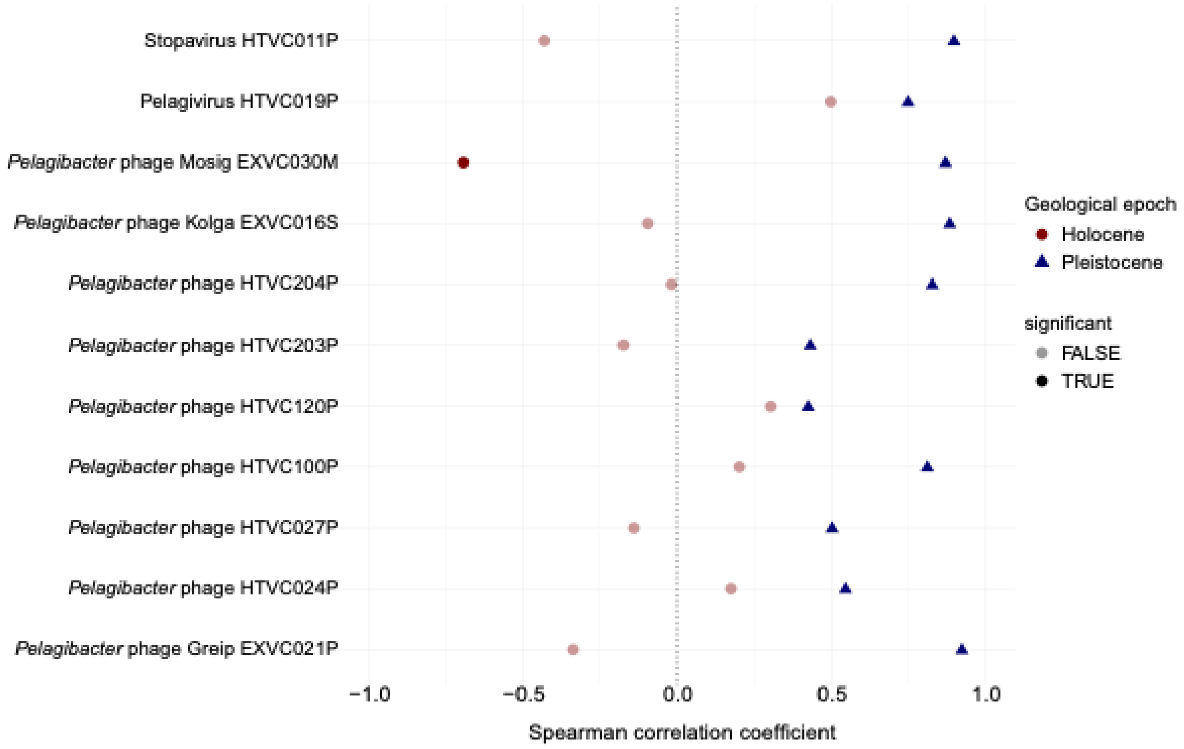

SFig. 16: Spearman correlation coefficients of relative abundances of *Pelagibacter*-infecting viruses and *Pelagibacter* strain IMCC9063 during the Pleistocene (blue triangles) and the Holocene (red circles). Significant correlations are shown in darker color. Host genus names contained within virus names are shown in italics for consistency.

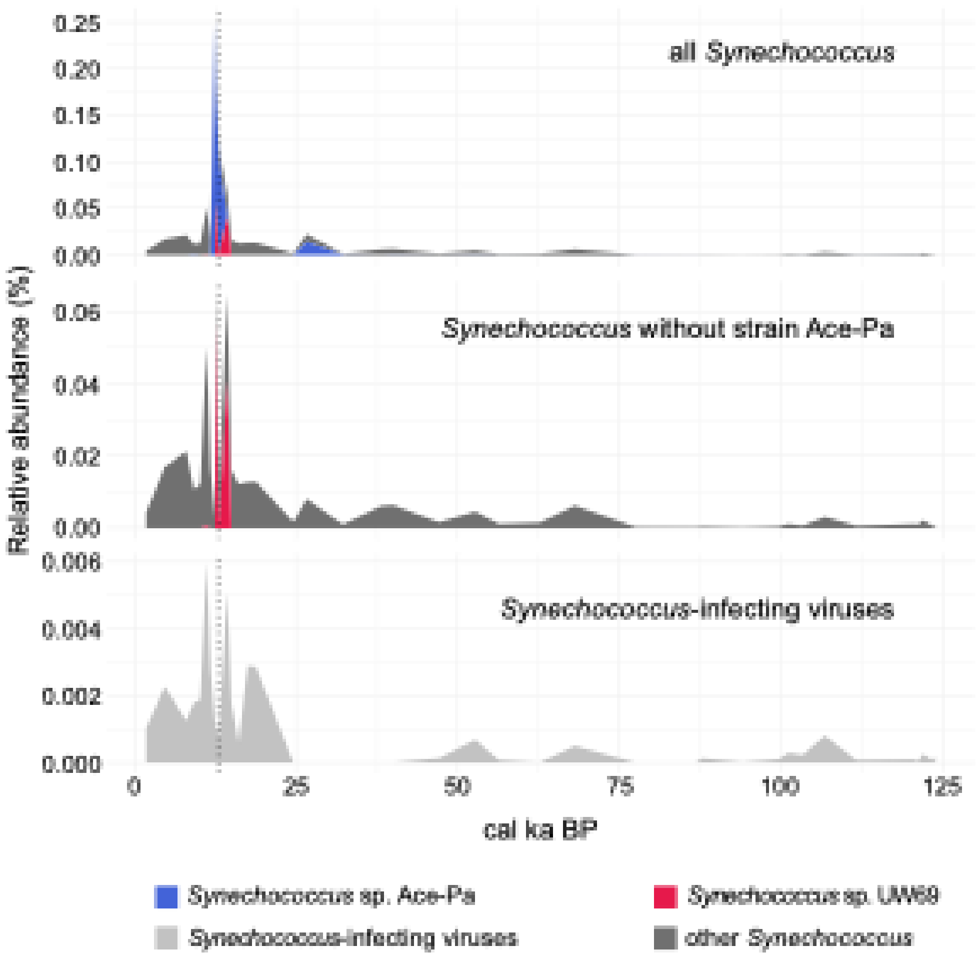

SFig. 17: Temporal patterns of *Synechococcus* and *Synechococcus*-infecting viruses in KL77. The upper plot shows all *Synechococcus* species and strains, with strain Ace-Pa highlighted in blue, strain UW69 highlighted in red and all others aggregated in dark grey. The middle plot displays *Synechococcus* species excluding strain Ace-Pa (dark grey), revealing temporal dynamics of less dominant taxa. The lower plot shows *Synechococcus*-infecting viruses (light grey). The dotted line indicates the timing of the Bering Strait opening.

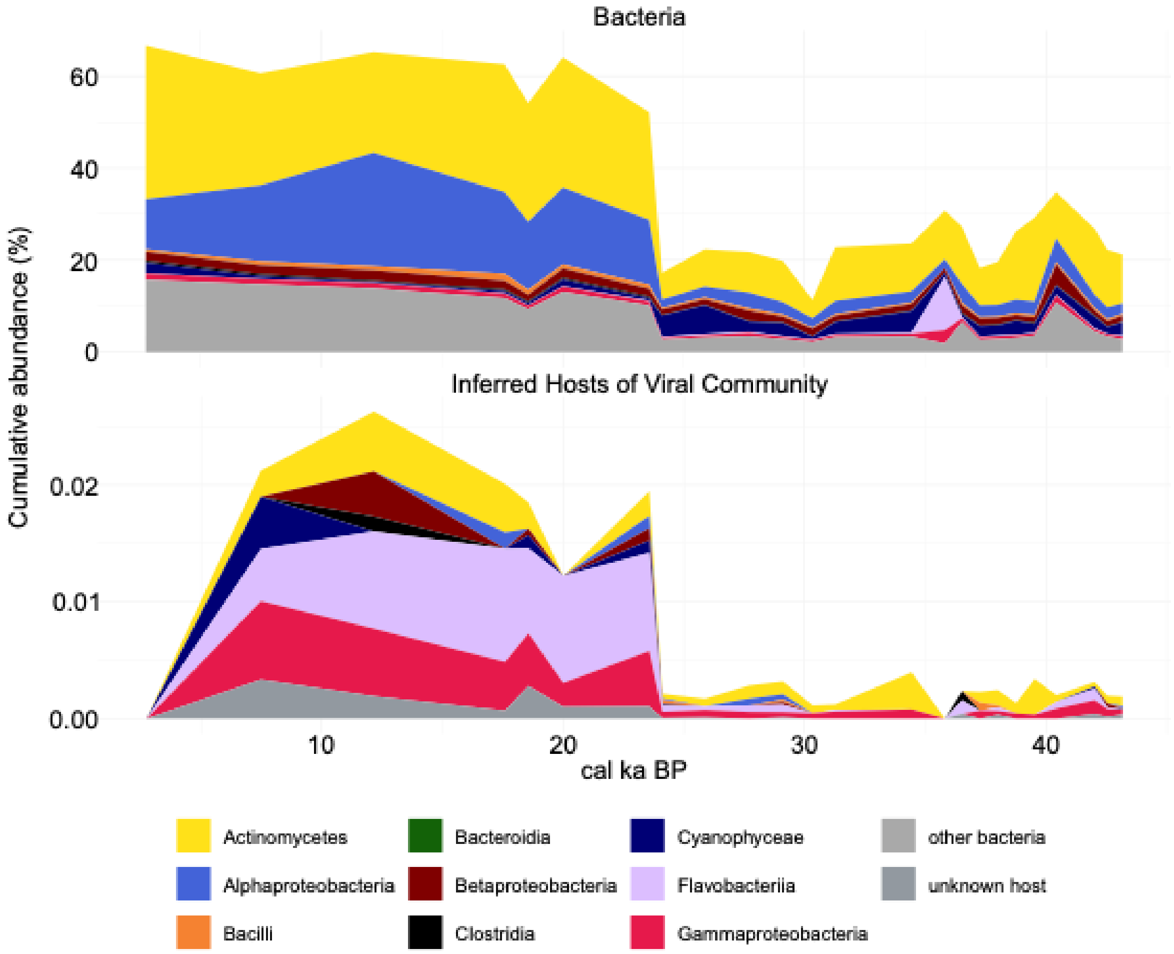

SFig. 18: Temporal patterns of bacteria and bacteria-infecting viruses in Lake Ulu. Colors indicate the bacterial class (upper plot) and the bacterial class infected by the respective viruses (lower plot).

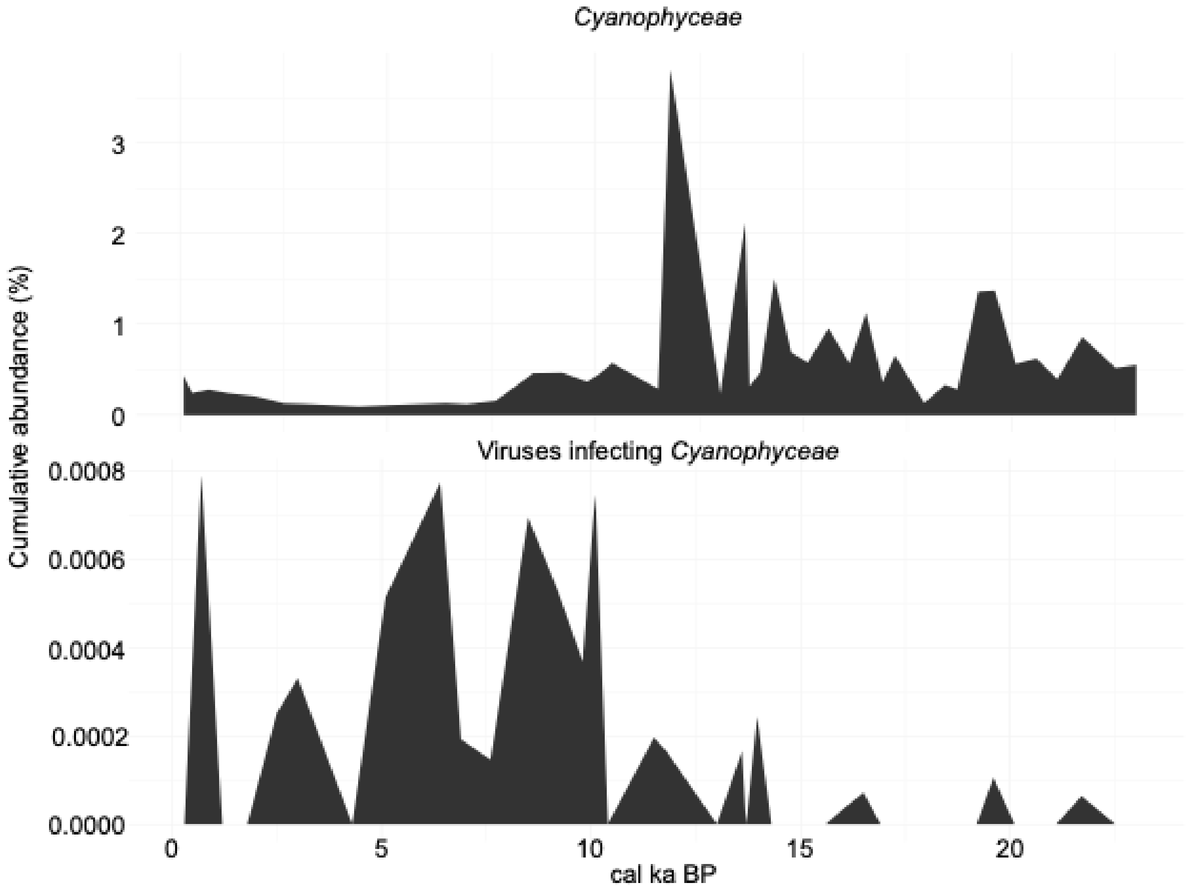

SFig. 19: Temporal patterns of *Cyanophyceae* and *Cyanophyceae*-infecting viruses in Lake Lama. The cumulative abundance is shown in respect to all mapped reads.

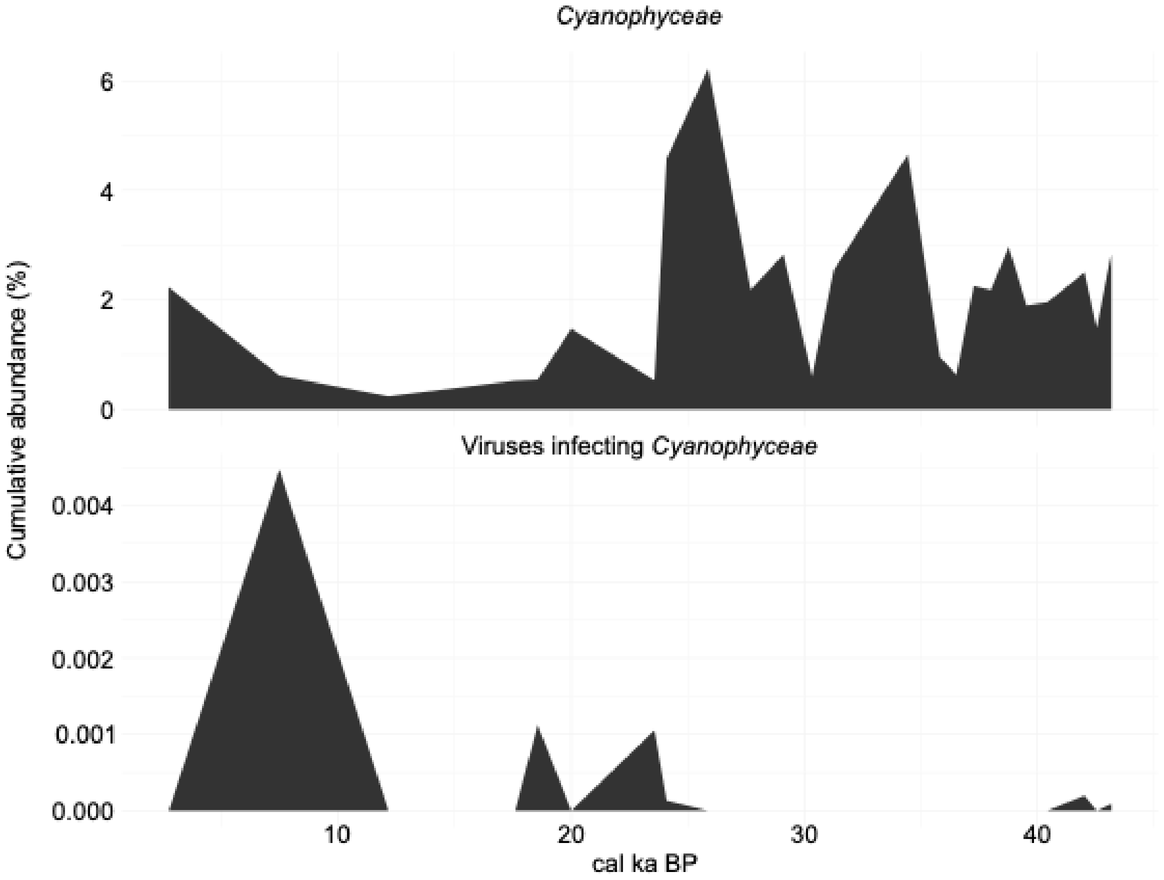

SFig. 20: Temporal patterns of *Cyanophyceae* and *Cyanophyceae*-infecting viruses in Lake Ulu. The cumulative abundance is shown in respect to all mapped reads.

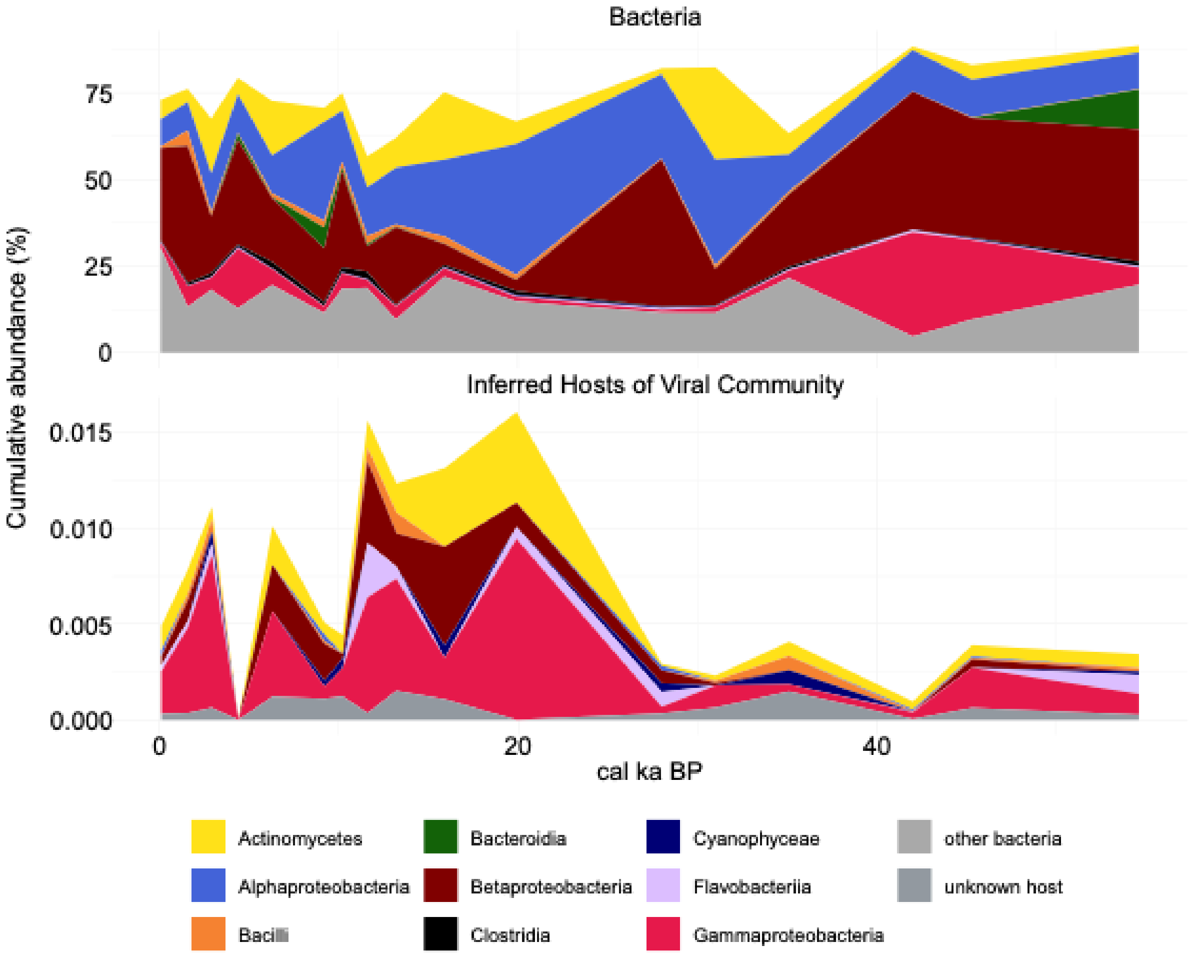

SFig. 21: Temporal patterns of bacteria and bacteria-infecting viruses in Lake Ilirney. Colors indicate the bacterial class (upper plot) and the bacterial class infected by the respective viruses (lower plot).

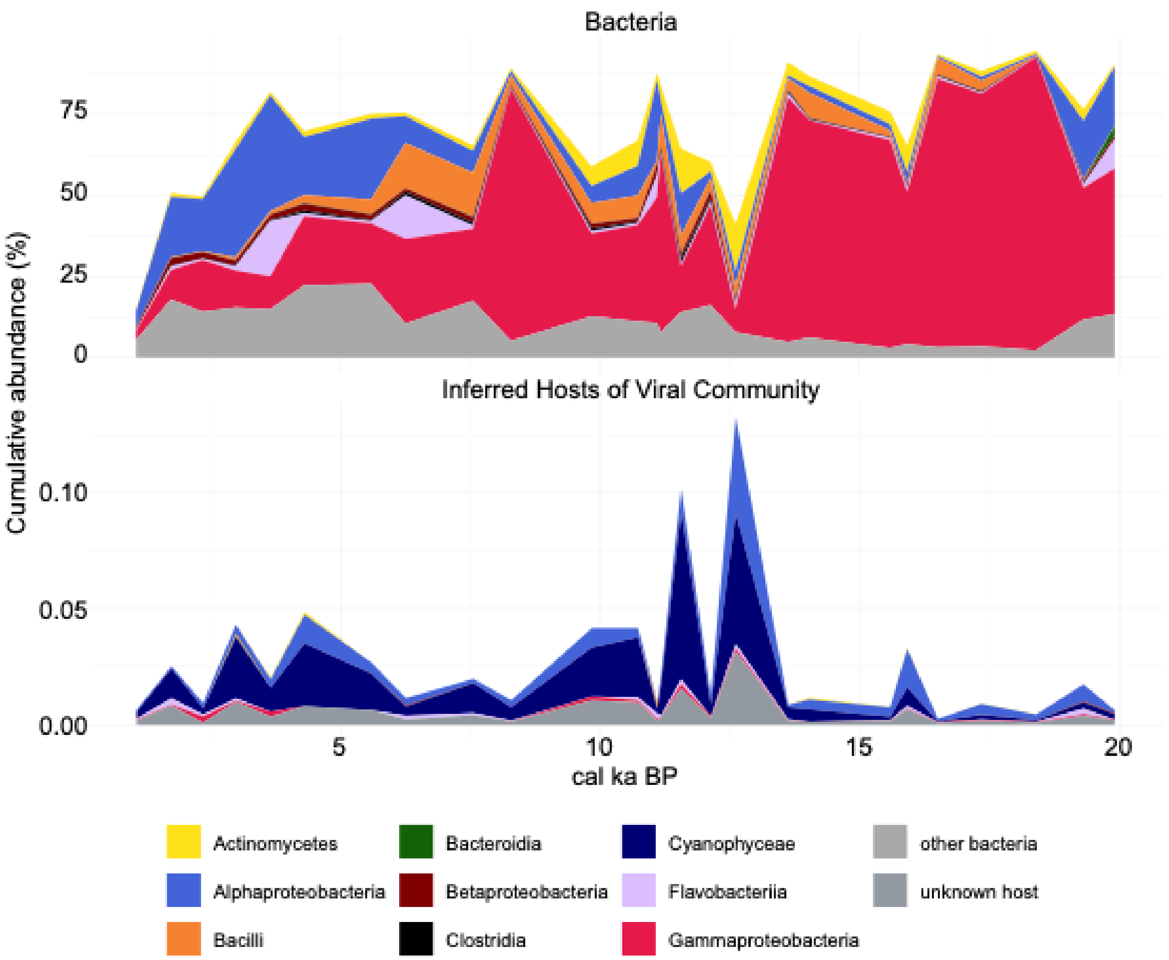

SFig. 22: Temporal patterns of bacteria and bacteria-infecting viruses in KL12. The cumulative abundance is shown in respect to all mapped reads. Colors indicate the bacterial class (upper plot) and the bacterial class infected by the respective viruses (lower plot).

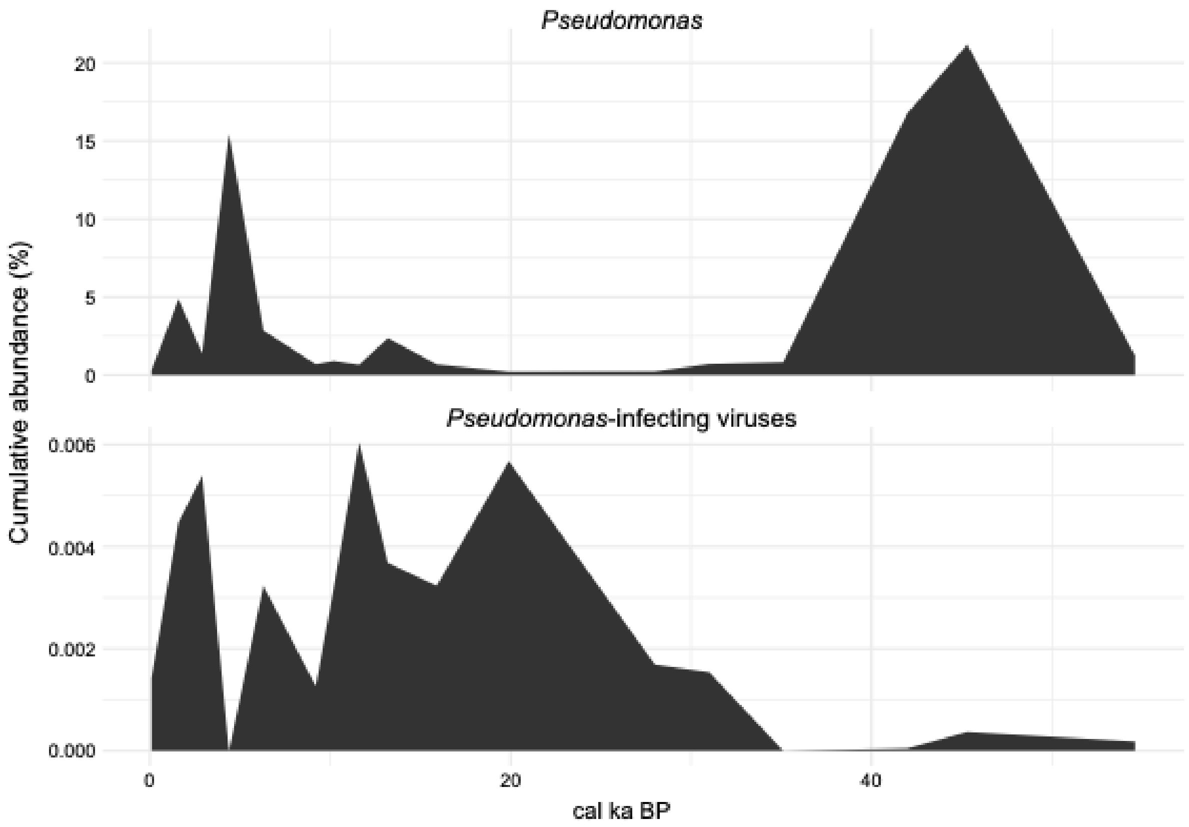

SFig. 23: Temporal patterns of *Pseudomonas* and *Pseudomonas*-infecting viruses in Lake Ilirney. The cumulative abundance is shown in respect to all mapped reads.

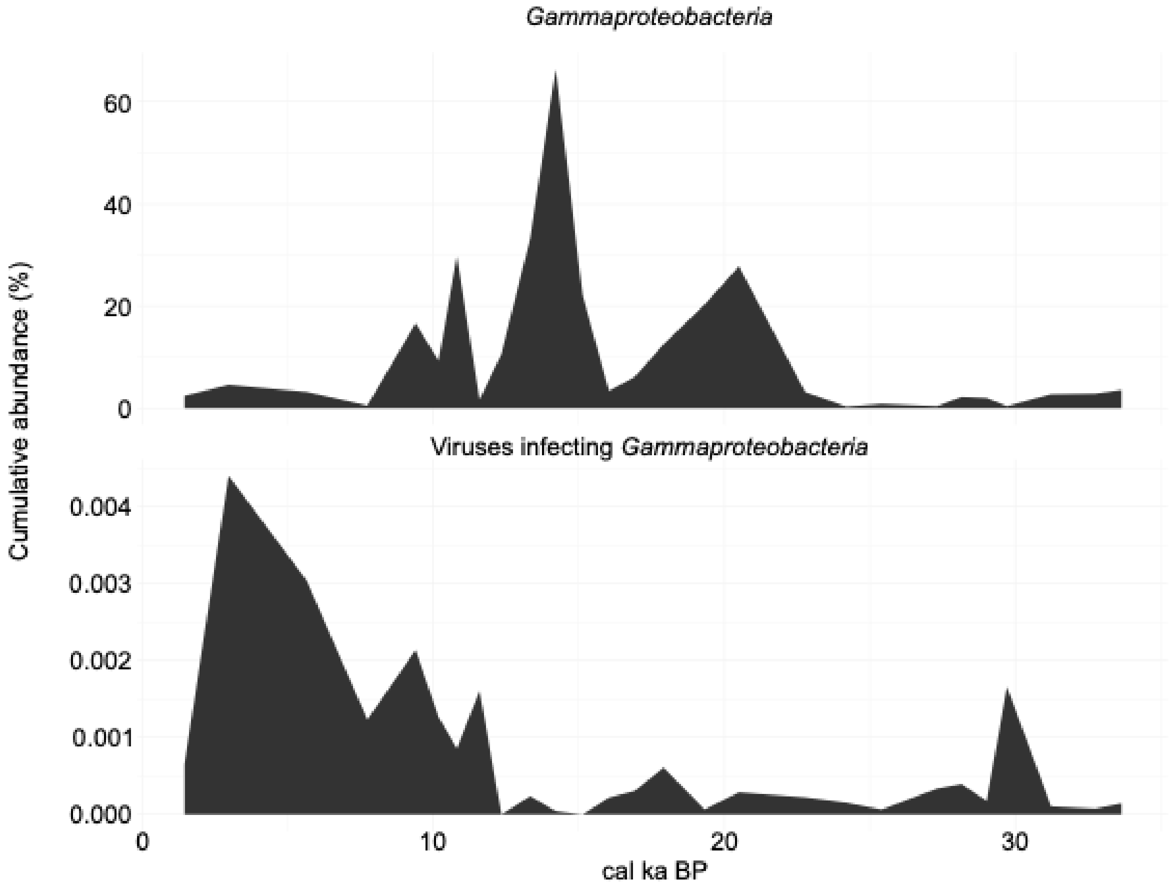

SFig. 24: Temporal patterns of *Gammaproteobacteria* and *Gammaproteobacteria*-infecting viruses in Lake Bolshoe Toko. The cumulative abundance is shown in respect to all mapped reads.

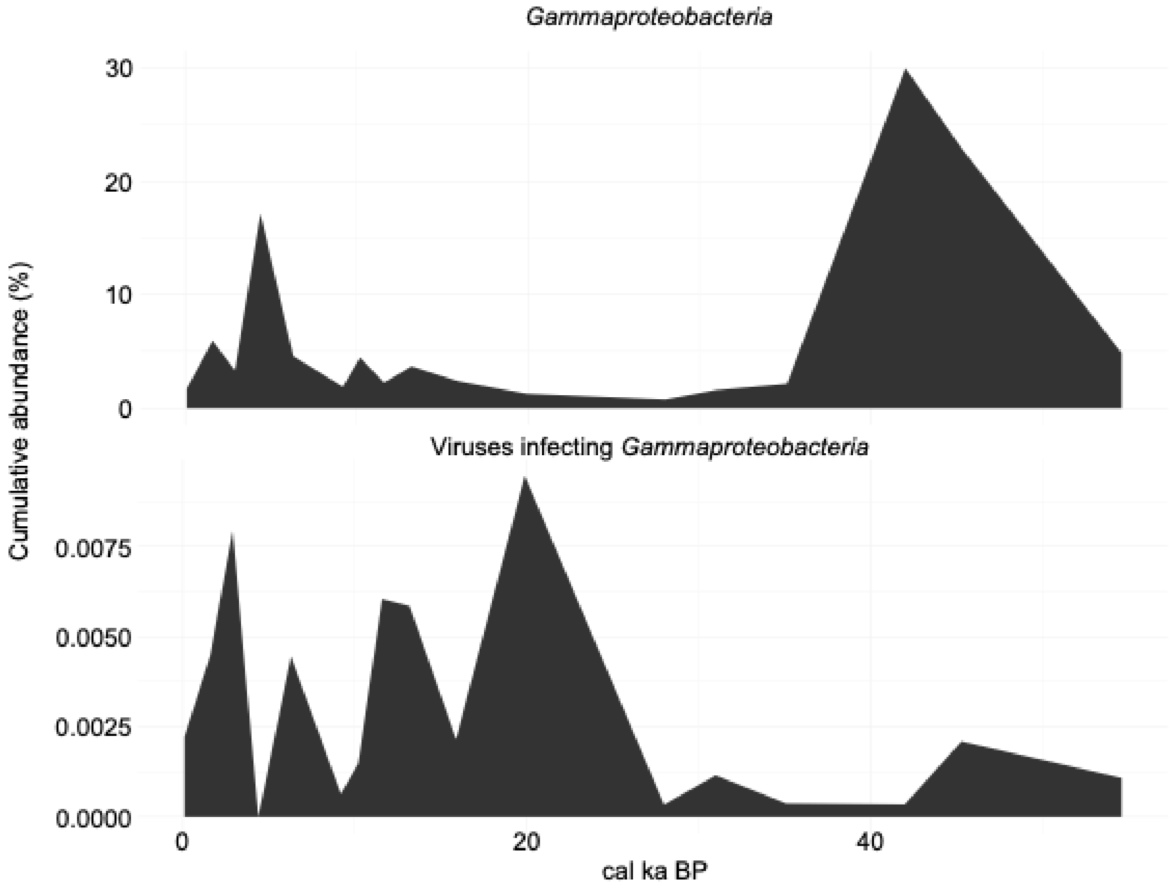

SFig. 25: Temporal patterns of *Gammaproteobacteria* and *Gammaproteobacteria*-infecting viruses in Lake Ilirney. The cumulative abundance is shown in respect to all mapped reads.

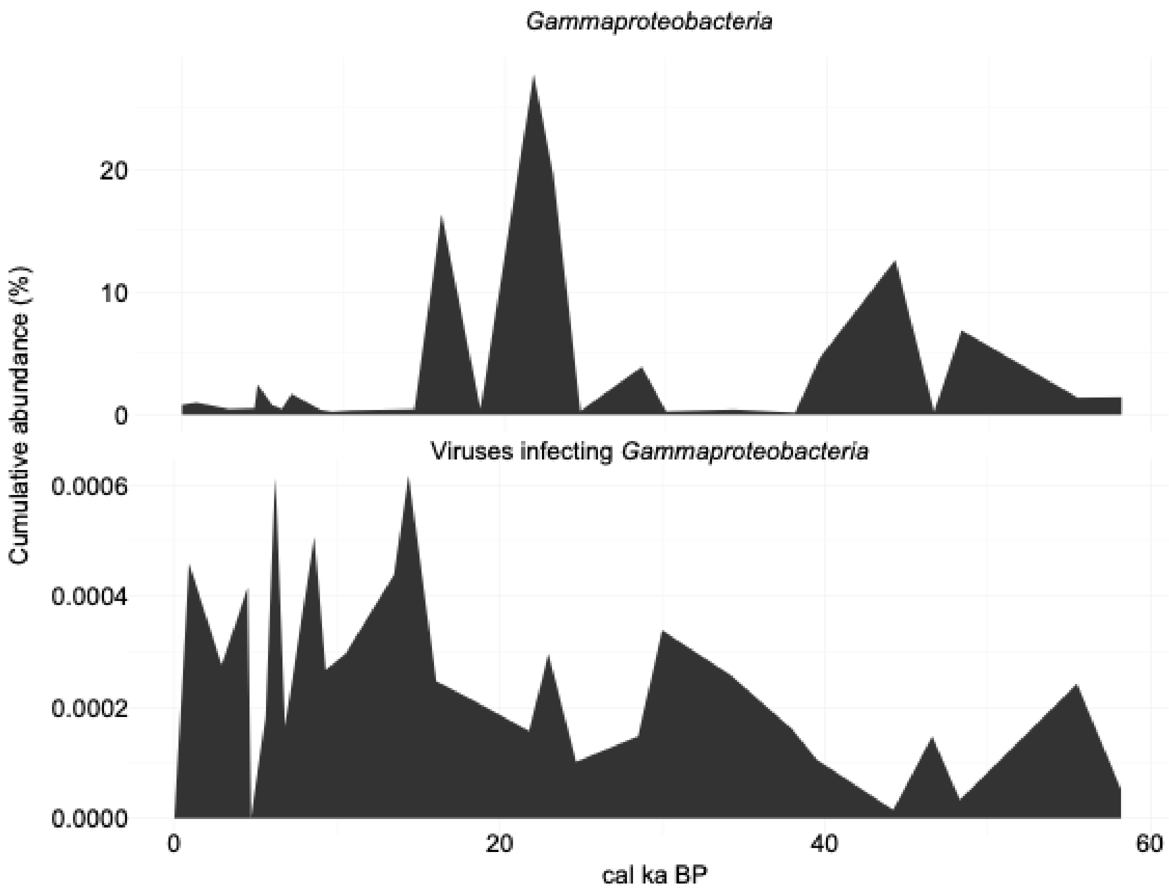

SFig. 26: Temporal patterns of *Gammaproteobacteria* and *Gammaproteobacteria*-infecting viruses in Lake Levinson-Lessing. The cumulative abundance is shown in respect to all mapped reads.

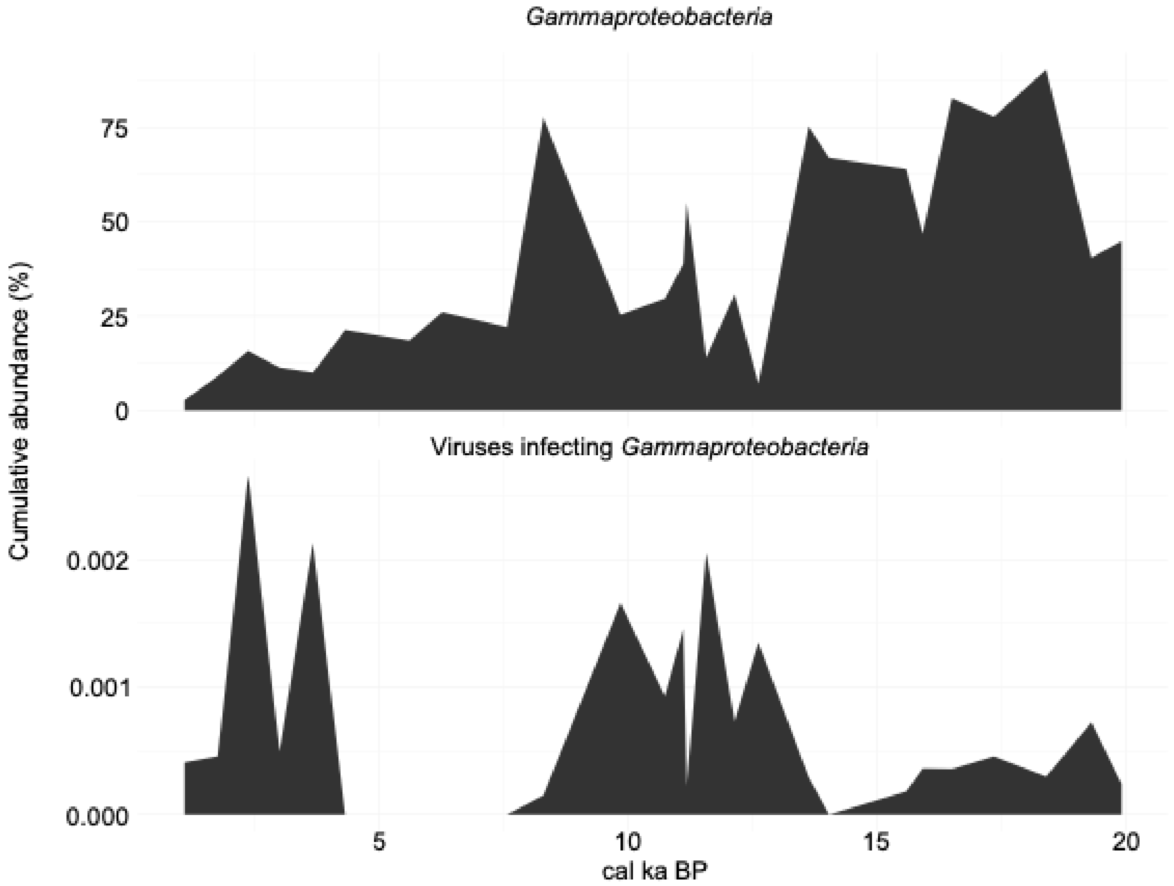

SFig. 27: Temporal patterns of *Gammaproteobacteria* and *Gammaproteobacteria*-infecting viruses in KL12. The cumulative abundance is shown in respect to all mapped reads.

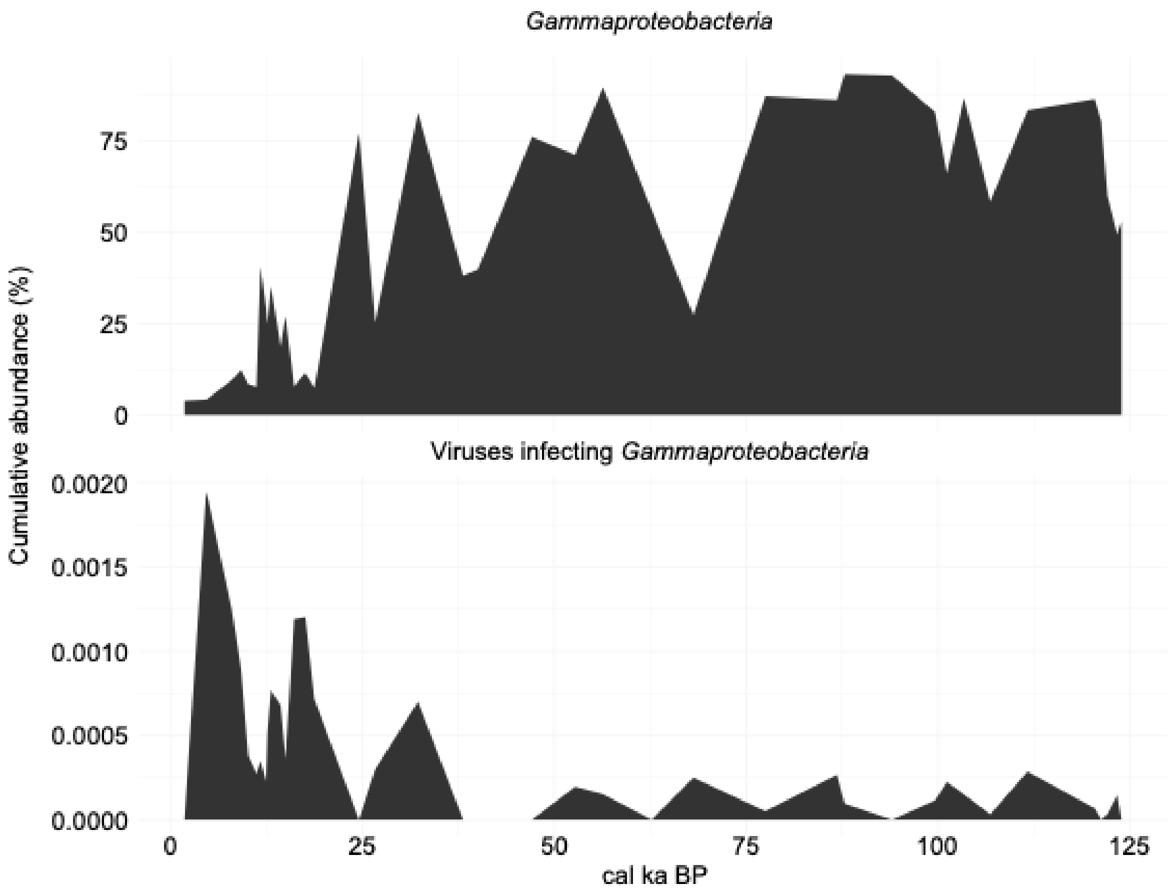

SFig. 28: Temporal patterns of *Gammaproteobacteria* and *Gammaproteobacteria*-infecting viruses in KL77. The cumulative abundance is shown in respect to all mapped reads.

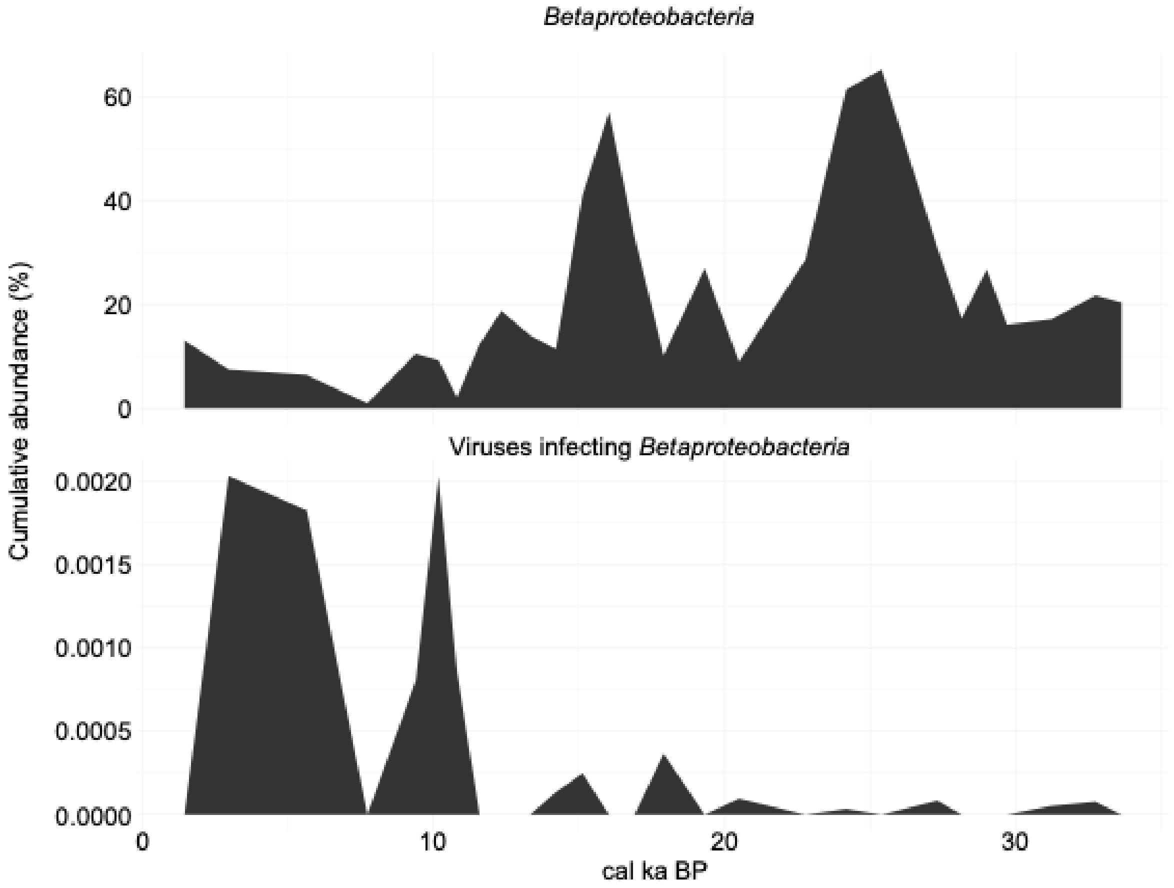

SFig. 29: Temporal patterns of *Betaproteobacteria* and *Betaproteobacteria*-infecting viruses in Lake Bolshoe Toko. The cumulative abundance is shown in respect to all mapped reads.

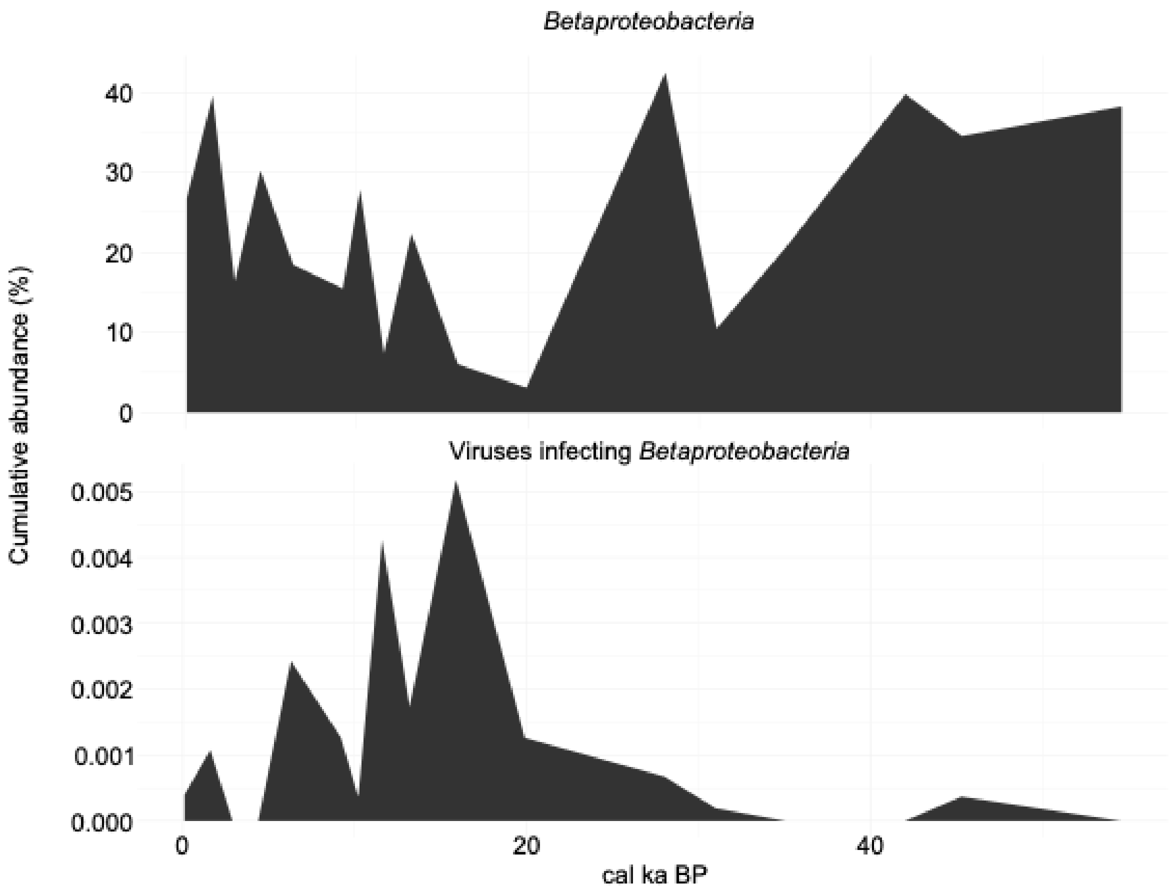

SFig. 30: Temporal patterns of *Betaproteobacteria* and *Betaproteobacteria*-infecting viruses in Lake Ilirney. The cumulative abundance is shown in respect to all mapped reads.
